# Abnormal mitochondrial iron metabolism damages alveolar type II epithelial cells involved in bleomycin-induced pulmonary fibrosis

**DOI:** 10.7150/thno.94072

**Published:** 2024-04-15

**Authors:** Min Shao, Haipeng Cheng, Xiaohong Li, Yujia Qiu, Yunna Zhang, Yanfen Chang, Jiafeng Fu, Mengxia Shen, Xinxin Xu, Dandan Feng, Yang Han, ShaoJie Yue, Yan Zhou, Ziqiang Luo

**Affiliations:** 1 Department of Physiology, Xiangya School of Medicine, Central South University, Changsha, Hunan 410078, China.; 2 Department of Pathology, The Second Xiangya Hospital, Central South University, Changsha, Hunan, 410000, China.; 3 Department of Pediatrics, Xiangya Hospital, Central South University, Changsha, Hunan, 410013, China.; 4 Hunan Key Laboratory of Organ Fibrosis, Changsha, Hunan, 410013, China.

**Keywords:** pulmonary fibrosis, type II alveolar epithelial cells, mitochondrial iron deposition, mitoferrin-2, F-box/LRR-repeat protein 5

## Abstract

**Rationale:** Pulmonary fibrosis is a chronic progressive lung disease with limited therapeutic options. We previously revealed that there is iron deposition in alveolar epithelial type II cell (AECII) in pulmonary fibrosis, which can be prevented by the iron chelator deferoxamine. However, iron in the cytoplasm and the mitochondria has two relatively independent roles and regulatory systems. In this study, we aimed to investigate the role of mitochondrial iron deposition in AECII injury and pulmonary fibrosis, and to find potential therapeutic strategies.

**Methods:** BLM-treated mice, MLE-12 cells, and primary AECII were employed to establish the mouse pulmonary fibrosis model and epithelial cells injury model, respectively. Mitochondrial transplantation, siRNA and plasmid transfection, western blotting (WB), quantitative real-time polymerase chain reaction (RT-qPCR), polymerase chain reaction (PCR), immunofluorescence, immunoprecipitation (IP), MitoSOX staining, JC-1 staining, oxygen consumption rate (OCR) measurement, and Cell Counting Kit-8 (CCK8) assay were utilized to elucidate the role of mitochondrial iron deposition in cell and lung fibrosis and determine its mechanism.

**Results:** This study showed that prominent mitochondrial iron deposition occurs within AECII in bleomycin (BLM)-induced pulmonary fibrosis mouse model and in BLM-treated MLE-12 epithelial cells. Further, the study revealed that healthy mitochondria rescue BLM-damaged AECII mitochondrial iron deposition and cell damage loss. Mitoferrin-2 (MFRN2) is the main transporter that regulates mitochondrial iron metabolism by transferring cytosolic iron into mitochondria, which is upregulated in BLM-treated MLE-12 epithelial cells. Direct overexpression of MFRN2 causes mitochondrial iron deposition and cell damage. In this study, decreased ubiquitination of the ubiquitin ligase F-box/LRR-repeat protein 5 (FBXL5) degraded iron-reactive element-binding protein 2 (IREB2) and promoted MFRN2 expression as well as mitochondrial iron deposition in damaged AECII. Activation of the prostaglandin E2 receptor EP4 subtype (EP4) receptor signaling pathway counteracted mitochondrial iron deposition by downregulating IREB2-MFRN2 signaling through upregulation of FBXL5. This intervention not only reduced mitochondrial iron content but also preserved mitochondrial function and protected against AECII damage after BLM treatment.

**Conclusion:** Our findings highlight the unexplored roles, mechanisms, and regulatory approaches of abnormal mitochondrial iron metabolism of AECII in pulmonary fibrosis. Therefore, this study deepens the understanding of the mechanisms underlying pulmonary fibrosis and offers a promising strategy for developing effective therapeutic interventions using the EP4 receptor activator.

## Introduction

Pulmonary fibrosis is a chronic and progressive lung disease characterized by increased fibrotic tissue, reduced lung compliance, impaired gas exchange, and respiratory insufficiency 1, 2. The median survival post-diagnosis typically ranges from 3 to 5 years. Although treatments such as Pirfenidone and Nintedanib help slow the progression of fibrosis, they are not curative 3-6. The type II alveolar epithelial cells (AECII) face extensive biosynthetic and metabolic challenges as a result of their dual roles: as progenitor cells and producers of surfactant phospholipids and proteins 7. The stain on AECII by malformed proteins, accumulated macromolecules, dysfunctional mitochondria, and foreshortened telomeres have been implicated in a number of chronic lung diseases, including pulmonary fibrosis 8. Previously, we observed aberrant iron deposition within the epithelial cells of patients with pulmonary fibrosis and mice subjected to bleomycin (BLM)-induced pulmonary fibrosis and administration of the iron chelator deferoxamine had the therapeutic potential for pulmonary fibrosis 9, 10. These findings indicate that imbalances in iron homeostasis within epithelial cells of lung tissue are involved in pulmonary fibrosis development. The labile iron pool of the cytoplasm, heme and iron-sulfur clusters of mitochondria, and lysosomes are the three main sites of intracellular iron existence 11. Coordinated iron allocation between intracellular compartments and the adaptation of iron uptake to intracellular regions are required for balanced iron homeostasis 12. Depending on cell type, mitochondria contain up to 20-50% of total cellular iron, and become the major site of intracellular iron utilization and regulation 13. Overload of iron in the labile cytoplasm iron pool is associated with reactive oxygen species (ROS) generation in the cytoplasm through the Fenton reaction, subsequently causing mitochondrial dysfunction to further induce ferroptosis 14-16. However, abnormal iron metabolism in mitochondria leads to too little or too much iron in the mitochondria, which directly leads to mitochondrial dysfunction and cell damage by alterations in Fe-S homeostasis, impaired mitochondrial dysfunction and increased mitochondrial oxidative stress 17. Notably, certain mitochondrial iron chelators exhibited a potent tumor growth suppressing activity compared to that in classical nonspecific chelators, suggesting that mitochondrial iron metabolism is independent of cytoplasmic iron metabolism and is essential for the regulation of cellular vital activities 18.

The accumulation of mitochondrial iron in erythroid precursors is known to trigger mitochondrial dysfunction, leading to ring sideroblasts and anemias 19, 20. Mitochondrial dysfunction increased mitochondrial ROS production, subsequently causing mitochondrial DNA (mtDNA) damage and release, reduced mitochondrial membrane potential, and ultimately induced cellular damage. When mitochondria dysfunction, mitochondrial transcription factor A (TFAM) loses its ability to wrap mtDNA entirely, akin to histones in nucleosomes, leading to mtDNA damage and release and subsequent cellular damage 21. Malfunctioning mitochondria have frequently been observed in AECII in the lungs of patients with idiopathic pulmonary fibrosis and mice with BLM-induced pulmonary fibrosis 22, 23. Concurrently, serum mtDNA levels in patients with idiopathic pulmonary fibrosis are positively correlated with disease severity 24.

Within the mitochondrion, metal ions contribute to the metalation, folding, and stability of many intrinsic mitochondrial proteins. Mitochondrial iron levels are intricately regulated by metallochaperones and metal transporters e.g., (mitoferrin-1 and mitoferrin-2 [MFRN2]) 25. MFRN2 functions as the principal mitochondrial iron transporter in non-erythroid cells 26, 27. Upregulated expression of MFRN2 was detected in the aortic endothelial cells of an atherosclerosis mouse model of atherosclerosis, and knockdown of this protein reduced endothelial dysfunction by decreasing mitochondrial iron levels 28. In addition, MFRN2 accumulation was detected in the brains of mice and humans with Huntington disease and correlated with reduced frataxin expression and mitochondrial bioenergetic dysfunction due to iron overload in the mitochondria of brain cells 29.

Moreover, iron-responsive element-binding protein 2 (IREB2), which regulates the transcription of cytoplasm iron transport-related genes, is an important iron-responsive element-binding protein in cells 30. Our experimental results revealed that protein content of IREB2 were substantially elevated in the AECII of BLM-induced pulmonary fibrosis mice. Furthermore, F-box/LRR-repeat protein 5 (FBXL5)-s-phase kinase-associated protein 1(SKP1)-cullin 1 (CUL1) complex formation is responsible for the ubiquitination and degradation of IREB2 and reduces the expression of downstream iron transport protein-related genes 31-33.

However, whether FBXL5 regulates IREB2 protein content and mitochondrial iron deposition in BLM-induced epithelial cell damage remains to be elucidated. Moreover, the role of MFRN2 in BLM-induced AECII damage remains unknown, and the role of MFRN2 and IREB2 in mitochondrial iron deposition and AECII damage remains unclear. Therefore, based on these above findings, we hypothesized that mitochondrial iron deposition promotes AECII damage and aggravates pulmonary fibrosis.

## Results

### Mitochondrial iron deposition in AECII during BLM-induced pulmonary fibrosis

**Figure 1:**
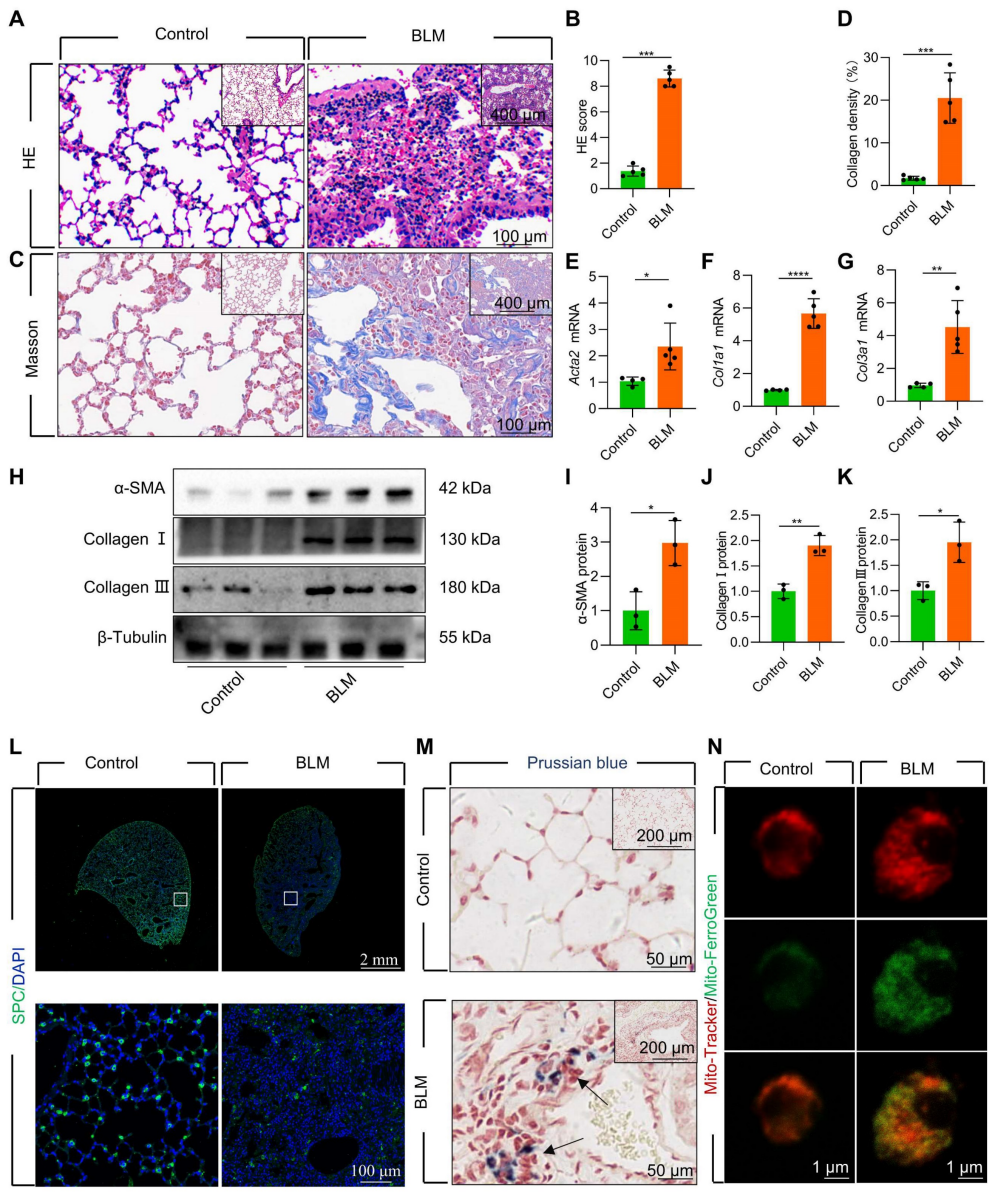
Mitochondrial iron deposition in AECII during single-dose BLM-induced pulmonary fibrosis. **(A)** Lung histopathology with HE staining was performed. **(B)** The HE score was evaluated by three blinded pathologists (*n* = 5 per group). **(C)** Masson's trichrome staining was employed to evaluate collagen disposition. **(D)** Quantification of the area occupied by fibrotic stroma, determined by Masson's trichrome staining (*n* = 5 per group). **(E-G)** mRNA levels of *Acta2*, *Col1a1*, and *Col3a1* in the lungs was detected using RT-qPCR (*n* = 4 - 5). **(H-K)** The protein levels of α-SMA, Collagen I, and Collagen III proteins in the lungs was detected using western blotting (*n* = 3). **(L)** SPC^+^ cells in healthy mice and pulmonary fibrosis mice were detected using an anti-SPC antibody (green) (Scale bar = 100 μm). **(M)** Iron deposition in lung tissues was assessed using Prussian blue staining. **(N)** Confocal detection of mitochondrial iron deposition in AECII from mice. *P < 0.05, ** *P* < 0.01, and *** *P* < 0.001.

We established a mouse model of pulmonary fibrosis via transtracheal single and repetitive-dose BLM. The severity of lung fibrosis and collagen deposition in BLM-induced mice was evaluated by using histological analysis with hematoxylin and eosin (HE) staining **(Figure 1A-B, Figure S1A-B)** and Masson's trichrome staining **(Figure 1C-D, Figure S1C-D)**. Additionally, we observed a considerable upregulation in the levels of fibrosis-related genes and proteins, including α-smooth muscle actin (α-SMA), Collagen I, and Collagen III **(Figure 1E-K, Figure S1E-K)**. Concomitant with the progression of fibrosis, we noted a substantial decrease in the number of surfactant protein C (SPC)-positive AECII in the lung tissue **(Figure 1L, Figure S1L)**. Moreover, the protein levels of SPC, a marker for AECII, were substantially reduced in pulmonary fibrosis **(Figure S2A-B)**, suggesting a pronounced loss of AECII in mice with BLM-induced lung fibrosis. Furthermore, we detected considerable iron deposition within the lung tissues of pulmonary fibrosis mice, corroborating earlier research findings 10 **(Figure 1M)**. To investigate the association between AECII injury and iron deposition, we isolated primary mice AECII from both pulmonary fibrosis and healthy lung tissues **(Figure S2C)**. Notably we discovered a marked iron accumulation within the mitochondria of AECII in pulmonary fibrosis tissues **(Figure 1N)**. This observation suggests that mitochondrial iron deposition in AECII could be a pivotal factor contributing to their damage and loss, ultimately promoting pulmonary fibrosis development.

### Mitochondrial iron deposition contributed to AECII injury after BLM-induced damage

**Figure 2:**
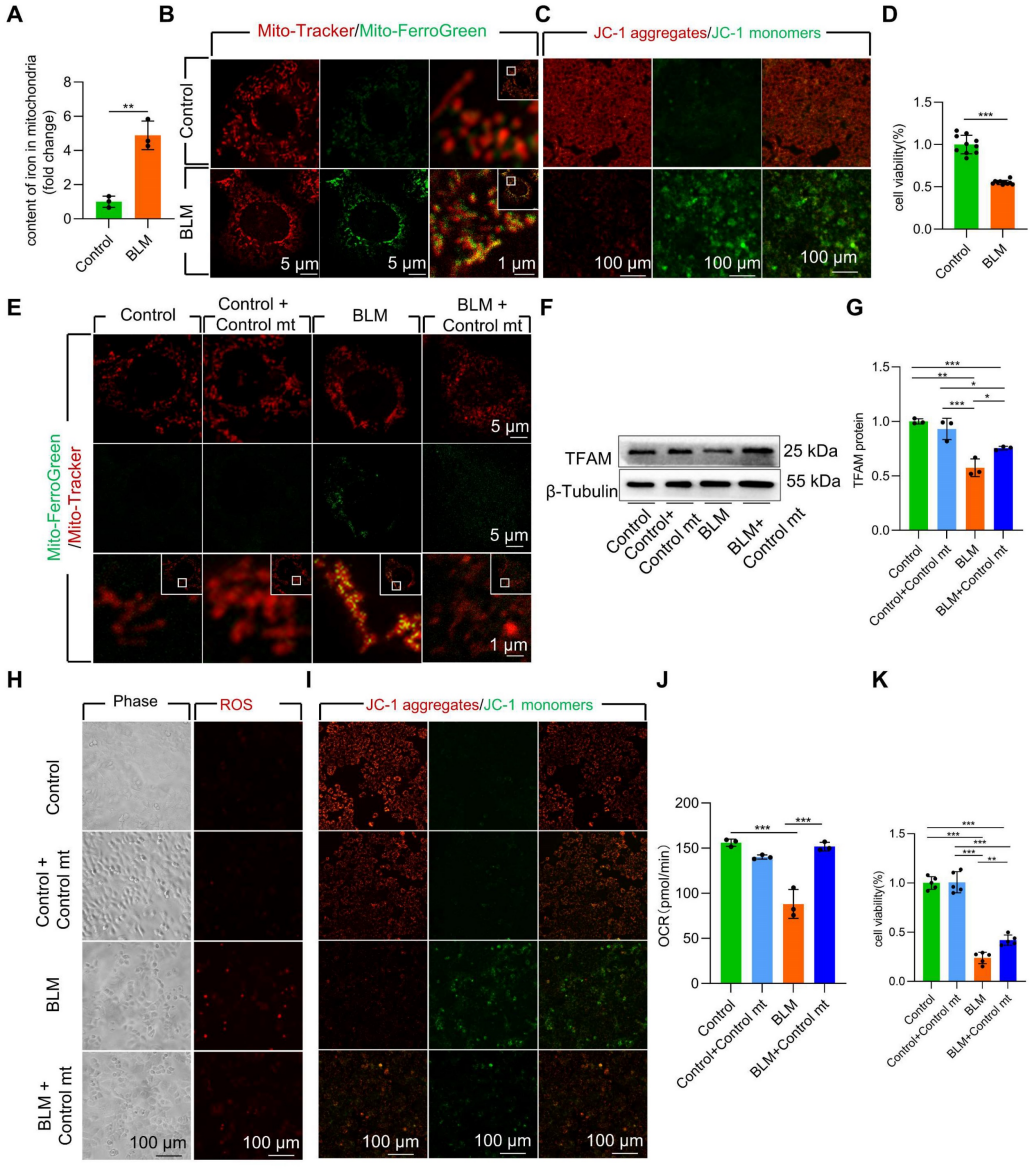
Mitochondrial iron deposition contributed to MLE-12 cells injury after BLM-induced damage. **(A)** Mitochondrial iron content was analyzed using an iron kit (*n* = 3). MLE-12 cells were treated with Mito-Tracker (red) and Mito-Ferro Green (green) and then imaged using a confocal microscope with Airyscan. **(B)** Representative images are shown (Scale bar = 5 μm), and boxes mark the enlarged images shown (Scale bar = 1 μm). **(C)** The Δψm in MLE-12 cells was measured using JC-1 staining (Scale bar = 100 μm). **(D)** MLE-12 cell viability was analyzed using CCK-8 (*n* = 10). **(E)** Healthy cell mitochondria were extracted was co-cultured with after 6 h BLM-treated MLE-12 cells and healthy MLE-12 cells. MLE-12 cells were stained with Mito-Tracker (red), and iron was stained with Mito-Ferro Green (green). Representative images are shown (Scale bar = 5 μm), and boxes mark the enlarged images shown (Scale bar = 1 μm). **(F-G)** TFAM protein levels in MLE-12 cells was detected using western blotting (*n* = 3). **(H)** ROS levels were analyzed using an ROS kit (Scale bar = 100 μm). **(I)** The Δψm in MLE-12 cells was measured using JC-1 staining (Scale bar = 100 μm). **(J)** Oxygen consumption rate (OCR) analysis of MLE-12 cells (*n* = 3). **(K)** MLE-12 cell viability was analyzed using CCK-8 (*n* = 5). *P < 0.05, ** *P* < 0.01, and *** *P* < 0.001.

To further study the role of mitochondrial iron deposition in AECII after BLM -induced damage, we utilized MLE-12 cells with AECII-like characteristics to establish an *in vitro* model of BLM-induced cell damage. We observed a considerable increase in both cellular and mitochondrial iron content **(Figure S3A, Figure 2A)** in BLM-treated MLE-12 cells, as quantified by mitochondrial extraction. Confocal imaging further supported these findings, revealing increased fluorescence intensity in iron-labeled mitochondria within BLM-damaged MLE-12 cells **(Figure 2B)**. Collectively, these results suggest that the mitochondrial iron deposition phenotype in BLM-induced MLE-12 cell injury closely resembles that observed in AECII from pulmonary fibrosis tissues.

Next, we conducted MitoSox Red staining to assess mitochondrial ROS levels and JC-1 staining to measure mitochondrial membrane potential (Δψm). A substantial increase in mitochondrial ROS content and a decline in Δψm in BLM-treated MLE-12 cells was observed **(Figure S3B, Figure 2C)**. Additionally, the viability of BLM-treated MLE-12 cells was considerably reduced **(Figure 2D)**. Collectively, these results indicate that BLM-injured MLE-12 cells with mitochondrial iron deposition have mitochondrial dysfunction.

To further validate the role of mitochondrial iron deposition in MLE-12 cell injury, we performed coculture experiments by combining BLM-injured MLE-12 cells with healthy mitochondria isolated from DsRed-labeled MLE-12 cells **(Figure S3C-E)**. Transplantation of healthy mitochondria considerably reduced mitochondrial iron deposition in BLM-treated MLE-12 cells **(Figure 2E)**. Moreover, healthy mitochondrial transplantation enhanced TFAM protein levels, thereby protecting mtDNA from damage **(Figure 2F-G)**. Furthermore, the transplantation of healthy mitochondria rescued mitochondrial function, as evidenced by the maintenance of the ROS content, Δψm and mitochondrial oxygen consumption rate (OCR) in the presence of iron deposition **(Figure 2H-J)**. Healthy mitochondrial transplantation successfully rescued cellular damage and prevented cell loss in BLM-induced MLE-12 cells **(Figure 2K)**. These findings underscore the critical role of mitochondrial iron deposition in BLM-induced dysfunction and damage in MLE-12 cells.

### MFRN2 promoted mitochondrial iron deposition in BLM-injured AECII

**Figure 3:**
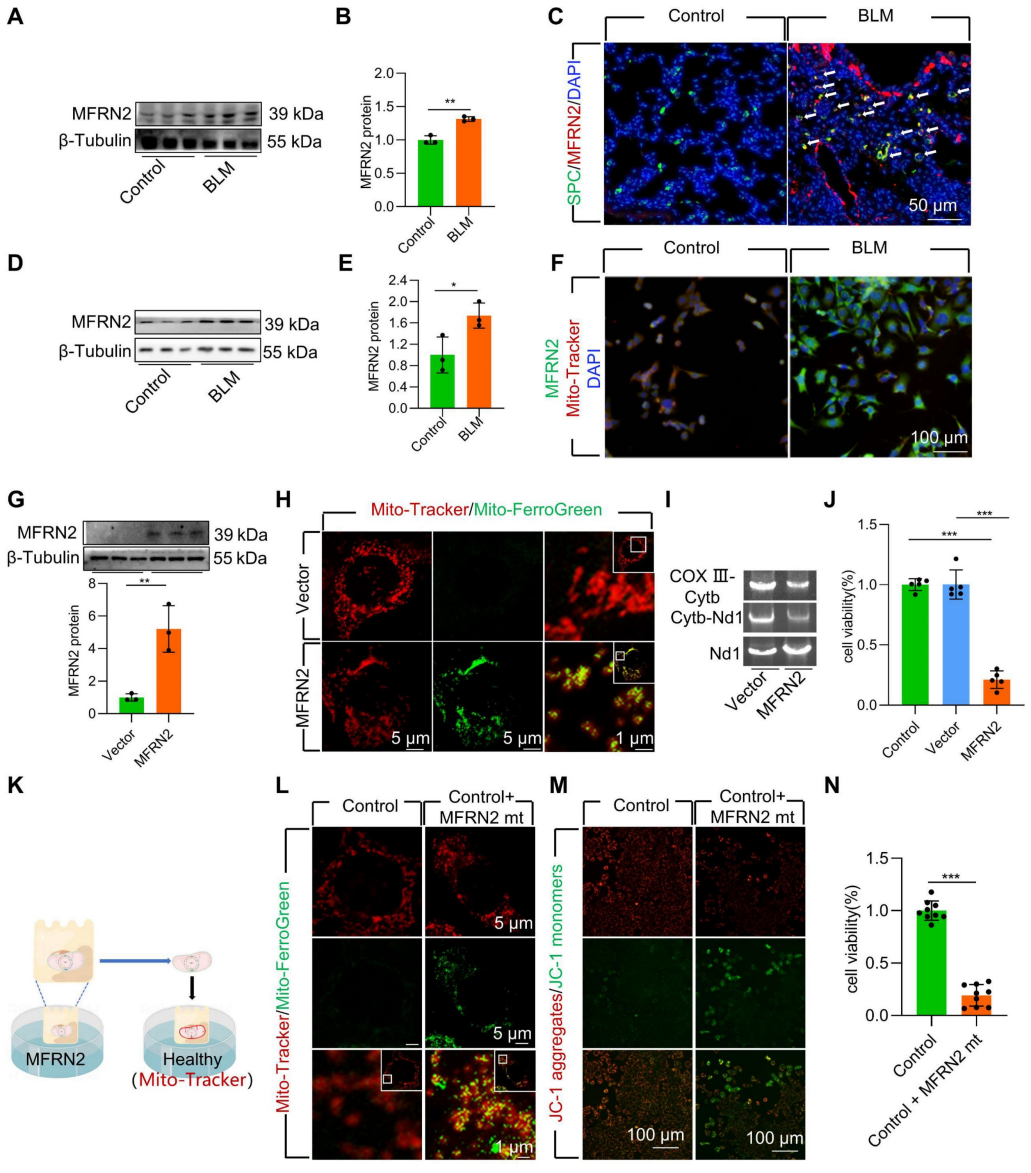
MFRN2 promoted mitochondrial iron deposition in single-dose BLM-induced pulmonary fibrosis in mice and BLM-induced damage in MLE-12 cells. **(A-B)** MFRN2 protein levels in the lungs was detected using western blotting (*n* = 3). **(C)** MFRN2 localization in AECII from control and pulmonary fibrosis mice was detected using anti-SPC antibodies (green) and anti-MFRN2 antibodies (red) (Scale bar = 50 μm). **(D-E)** MFRN2 protein levels in MLE-12 cells was detected using western blotting (*n* = 3). **(F)** MFRN2 localization in the mitochondria of control and BLM-injured MLE-12 cells was determined using anti-MFRN2 antibodies (green) and Mito-Tracker (red) (Scale bar = 100 μm). **(G)** MFRN2 protein levels in MLE-12 cells was detected using western blotting (*n* = 3). **(H)** MLE-12 cells were stained with Mito-Tracker (red) and Mito-Ferro Green (green) and then imaged using confocal microscopy with Airyscan. Representative images are shown (Scale bar = 5 μm), and boxes mark the enlarged images shown (Scale bar =1 μm). **(I)** The two mitochondrial DNA fragments Cytb-Nd1 and COX III-Cytb were detected using agarose gel electrophoresis. **(J)** MLE-12 cell viability was analyzed using CCK-8 (*n* = 5). **(K)** MFRN2 overexpressing mitochondria were extracted was cocultured with healthy MLE-12 cells. **(L)** MLE-12 cells were stained with Mito-Tracker (red), and the iron was stained with Mito-Ferro Green (green). Representative images are shown (Scale bar = 5 μm), and boxes mark the enlarged images shown (Scale bar = 1 μm). **(M)** The Δψm in MLE-12 cells was measured using JC-1 staining (Scale bar = 100 μm). **(N)** MLE-12 cell viability was analyzed using CCK-8 (*n* = 9). *P < 0.05, ** *P* < 0.01 and *** *P* < 0.001.

To further explore the mechanism underlying mitochondrial iron deposition in BLM-injured AECII, we examined changes in MFRN2 expression, a pivotal mediator of iron transport into the mitochondria. Our investigation revealed a considerable increase in the protein levels MFRN2 during BLM-induced pulmonary fibrosis in mice **(Figure 3A-B, Figure S4A-B)**. Simultaneously, dual staining of MFRN2 with SPC demonstrated a substantial upregulation of MFRN2 protein levels, specifically within AECII of mice exposed to BLM **(Figure 3C, Figure S4C)**. However, dual staining of MFRN2 with AGER demonstrated that the number of MFRN2^+^ alveolar epithelial type Ⅰ cell (AECⅠ) did not significantly increase in pulmonary fibrosis mice compared with that in the control mice **(Figure S5)**. Concurrently, the increased MFRN2 levels were further confirmed by western blotting and immunofluorescence in BLM-treated MLE-12 cells **(Figure 3D-F)**.

To ascertain the pivotal role of MFRN2 in inducing mitochondrial iron deposition and AECII injury and loss, we transfected MLE-12 cells with an MFRN2-overexpression plasmid. We observed that MFRN2 overexpression substantially enhanced mitochondrial iron deposition, leading to mtDNA damage **(Figure 3G-I)**. Furthermore, MFRN2 overexpression decreased cell viability **(Figure 3J)**.

To support these findings, we conducted coculture experiments involving healthy cells and mitochondria isolated from MLE-12 cells overexpressing MFRN2 **(Figure 3K)**. MFRN2-overexpressing mitochondria induced iron deposition in healthy mitochondria **(Figure 3L)**. Concurrently, the Δψm of the healthy MLE-12 cells exhibited a considerable decrease in response to MFRN2-overexpressing mitochondria **(Figure 3M)**. Additionally, the viability of healthy MLE-12 cells was substantially reduced upon exposure to mitochondria overexpressing MFRN2 **(Figure 3N)**.

Collectively, these findings underscore the role of MFRN2 in promoting mitochondrial iron deposition, which ultimately contributes to BLM-induced dysfunction and damage in MLE-12 cells.

### FBXL5 regulated the IREB2-MFRN2 axis, attenuating mitochondrial iron deposition and protecting AECII from BLM-induced injury

**Figure 4:**
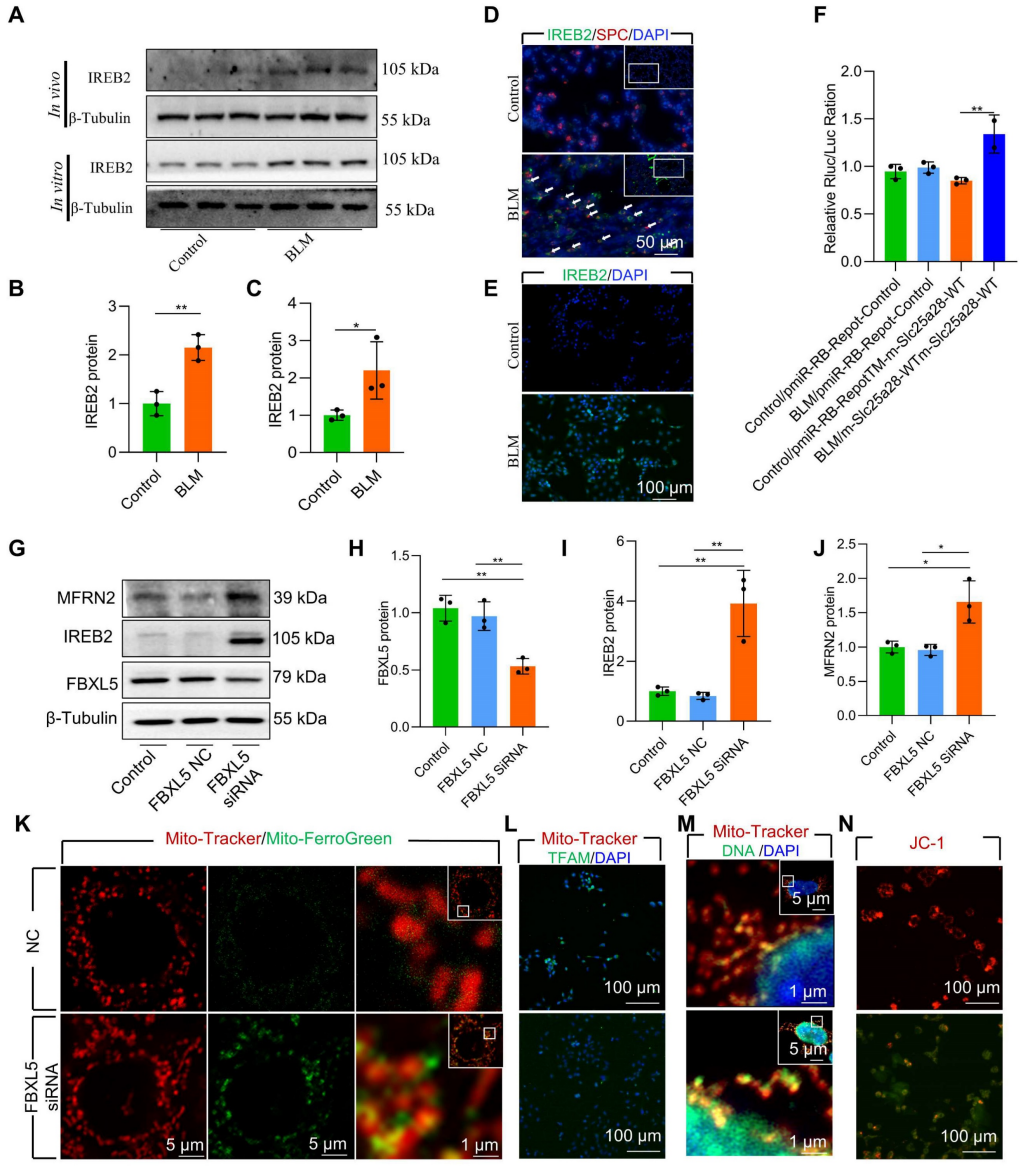
FBXL5 regulated the IREB2-MFRN2 axis, attenuating mitochondrial iron deposition and protecting AECII cells from single-dose BLM-induced injury and MLE-12 cells from BLM damage. **(A-B)** IREB2 protein levels in the lungs of mice was detected using western blotting (*n* = 3). **(A and C)** IREB2 protein levels in MLE-12 cells was detected using western blotting (*n* = 3). **(D)** IREB2 localization in AECII of the control and pulmonary fibrosis mice was determined using anti-IREB2 antibodies (green) and anti-SPC antibodies (red) (Scale bar = 50 μm). **(E)** IREB2 protein levels in MLE-12 cells was detected using immunofluorescence staining (Scale bar = 100 μm). **(F)** The interaction between IREB2 and MFRN2 was assessed by dual luciferase reporter gene assay (*n* = 3). **(G-J)** FBXL5, IREB2, and MFRN2 protein levels in MLE-12 cells was detected using western blotting (*n* = 3). **(K)** MLE-12 cells were stained with Mito-Tracker (red), and iron was stained with Mito-Ferro Green (green), and then they were imaged using confocal microscopy with Airyscan. Representative images are shown (Scale bar = 5 μm), and boxes mark the enlarged images shown (Scale bar = 1 μm). **(L)** TFAM protein levels in MLE-12 cells was detected using immunofluorescence staining (Scale bar = 100 μm). **(M)** MLE-12 cells were transduced with Mito-Tracker (red), and the DNA was transduced with anti-DNA antibody (green) and then imaged by confocal microscopy with Airyscan. Representative images are shown (Scale bar = 1 μm), and boxes mark the enlarged images shown (Scale bar = 1 μm). **(N)** The Δψm in MLE-12 cells was measured using JC-1 staining (Scale bar = 100 μm). * P < 0.05, ** *P* < 0.01.

STRING analysis revealed a mutual regulatory relationship between IREB2 and MFRN2 **(Figure S6A)**. Furthermore, elevated IREB2 levels were observed in AECII of BLM-induced mice and in BLM-treated MLE-12 cells **(Figure 4A-E, Figure S7)**. To further verify the regulation effect of IREB2 on MFRN2, we identified binding sites between IREB2 and 3′- untranslated region (UTR)-MFRN2 through database analysis (http://service.tartaglialab.com/page/catrapid_group). To validate the sequence-specific nature of this interaction and its involvement in BLM-induced AECII injury, we constructed wild-type and pmiR-RB-REPORT™ 3′-UTR reporter vectors. Dual luciferase results confirmed that BLM treatment considerably increased the luciferase activity of both IREB2 and MFRN2, indicating 3′-UTR-MFRN2 as a target of IREB2 **(Figure 4F)**. These data suggest that upregulation of IREB2 promotes MFRN2 expression in BLM-injured MLE-12 cells.

FBXL5 negatively regulates the expression of intracellular iron metabolism-related genes by promoting the ubiquitination and degradation of IREB2 34. FBXL5 protein levels in AECII are substantial reduced in both BLM-induced pulmonary fibrosis mouse lung tissue and BLM-12 cells damaged by BLM **(Figure S6B-C)**. To directly demonstrate the effect of FBXL5 dropping in mitochondrial iron deposition, we used small interfering RNA (siRNA) for knockdown of FBXL5. The knockdown of FBXL5 using siRNA considerably increased IREB2 and MFRN2 protein levels in MLE-12 cells **(Figure 4G-J)**. Additionally, FBXL5 knockdown substantially promoted mitochondrial iron deposition in MLE-12 cells, as confirmed by confocal microscopy **(Figure 4K)**. Immunofluorescence showed that FBXL5 knockdown considerably decreased TFAM protein levels **(Figure 4L)**. Furthermore, FBXL5 knockdown substantially promoted mtDNA release **(Figure 4M)**. The Δψm of healthy mitochondria was considerably decreased in response to FBXL5 knockdown in MLE-12 cells **(Figure 4N)**. These findings suggest that the IREB2-MFRN2 signaling pathway is activated in FBXL5-knockdown MLE-12 cells.

Collectively, these data indicate that the IREB2-MFRN2 axis, regulated by FBXL5, plays a protective role against BLM-induced mitochondrial iron deposition in AECII.

### Activation of EP4 receptor improved mitochondrial iron deposition in AECII, mitigating injury following BLM exposure

**Figure 5:**
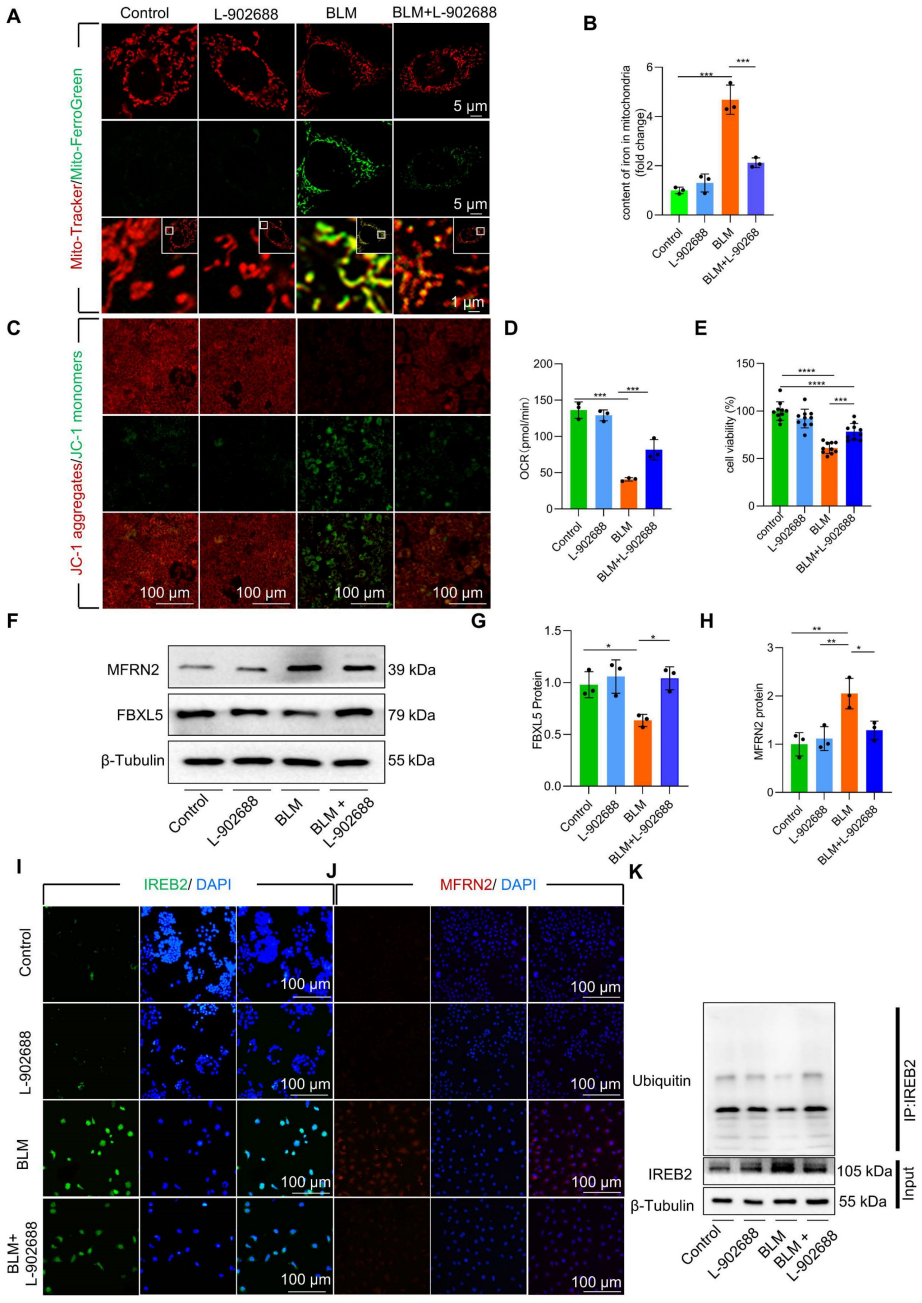
Activation of the EP4 receptor improved mitochondrial iron deposition in MLE-12 cells, mitigating injury following BLM exposure. **(A)** MLE-12 cells were transduced with Mito-Tracker (red), and iron was transduced with Mito-Ferro Green (green) and then imaged using confocal microscopy with Airyscan. Representative images are shown (Scale bar = 5 μm), and boxes mark the enlarged images shown (Scale bar = 1 μm). **(B)** Mitochondrial iron content was analyzed using an iron kit (*n* = 3).** (C)** The Δψm in MLE-12 cells was measured using JC-1 staining (Scale bar = 100 μm). **(D)** Oxygen consumption rate (OCR) analysis of MLE-12 (J, *n* = 3). **(E)** MLE-12 cell viability was analyzed using CCK-8 (*n* = 10). **(F-H)** FBXL5 and MFRN2 protein levels in MLE-12 cells was detected using western blotting (*n* = 3). **(I)** IREB2 protein levels in MLE-12 cells was detected using immunofluorescence staining (Scale bar = 100 μm). **(J)** MFRN2 protein levels in MLE-12 cells was detected using immunofluorescence staining (Scale bar = 100 μm). **(K)** IREB2 ubiquitination was analyzed using Co-IP. * *P* < 0.05, ** *P* < 0.01, *** *P* < 0.001.

Although the role of EP4 as a prostaglandin E2 (PGE2) receptor in anti-inflammatory processes has been extensively observed, its involvement in pulmonary fibrosis remains unclear. To explore effective treatment strategies for alleviating mitochondrial iron deposition, we employed the EP4 receptor agonist L-902688 in MLE-12 cells. Activation of the EP4 receptor substantially reduced mitochondrial iron content, as observed by confocal microscopy and mitochondrial extraction **(Figure 5A-B)**. Furthermore, EP4 receptor activation considerably preserved Δψm and mitochondrial dysfunction in MLE-12 cells and primary AECII exposed to BLM, as demonstrated by JC-1 staining and OCR measurements **(Figure 5C-D, Figure S8A)**. Moreover, the viability of MLE-12 cells and primary AECII exposed to BLM substantially increased upon EP4 receptor activation, as assessed by using the Cell Counting Kit-8 (CCK8) assay **(Figure 5E, Figure S8B)**.

We further investigated whether EP4 receptor activation regulated FBXL5, a key factor in regulating mitochondrial iron deposition. Notably, we found that EP4 receptor activation substantially increased FBXL5 protein levels and reduced IREB2 and MFRN2 protein levels in BLM-treated MLE-12 cells and primary AECII **(Figure 5F-J, Figure S8C-E)**. Furthermore, EP4 receptor activation promoted the ubiquitination of IREB2 in BLM-treated MLE-12 cells, as demonstrated by co-immunoprecipitation **(**Co-IP; **Figure 5K)**.

These findings suggest that EP4 receptor activation improves mitochondrial iron deposition and cellular injury in BLM-treated AECII.

### Activation of the EP4 receptor improved BLM-induced mitochondrial iron deposition via FBXL5 regulation of the IREB2-MFRN2 axis in MLE-12 cells

**Figure 6:**
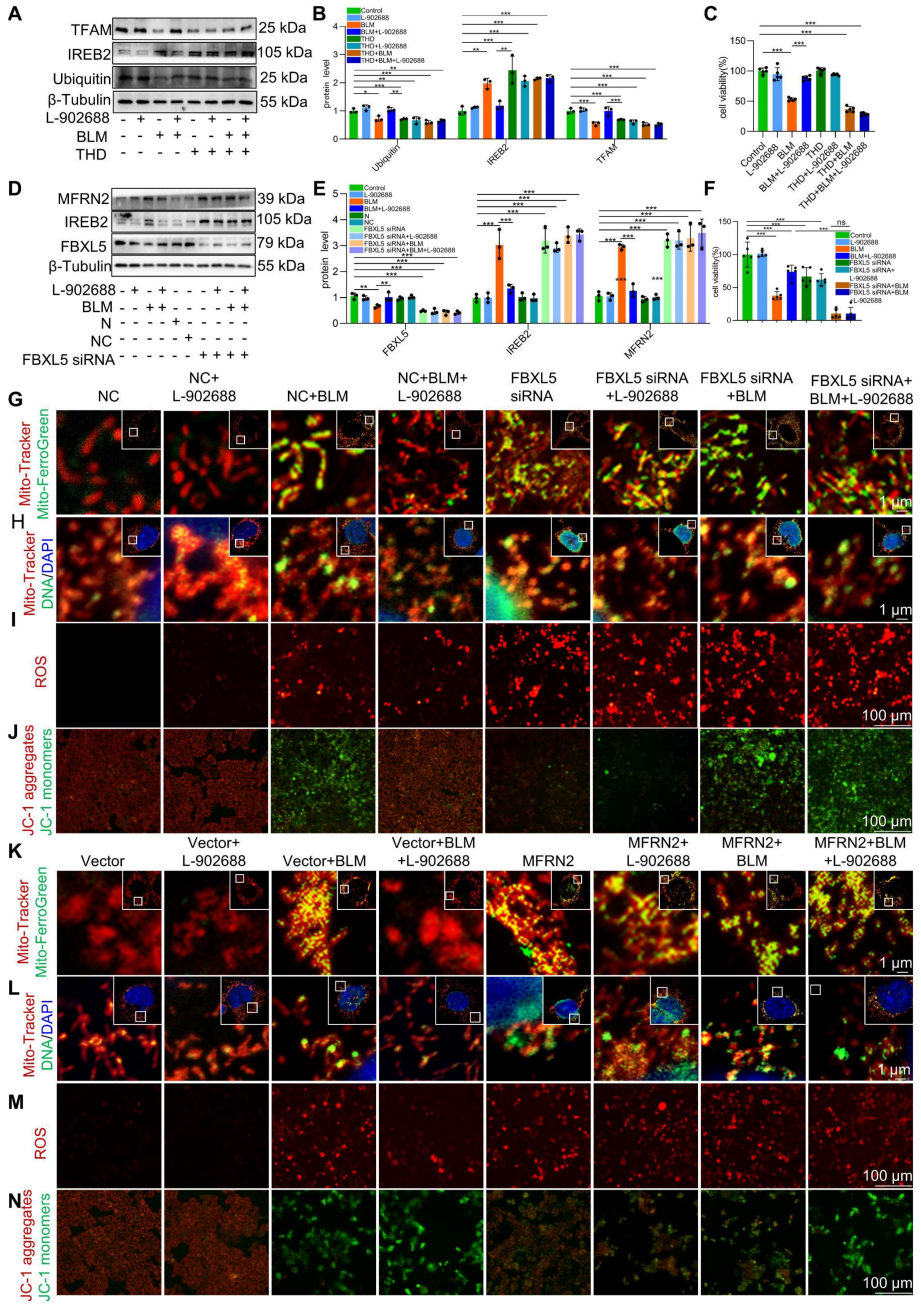
Activation of the EP4 receptor improved BLM-induced mitochondrial iron deposition via FBXL5 regulation of the IREB2-MFRN2 axis in MLE-12 cells. **(A-B)** Ubiquitin, IREB2, and TFAM protein levels in MLE-12 cells was detected using western blotting (*n* = 3). **(C)** MLE-12 cell viability was analyzed using CCK-8 (*n* = 5). **(D-E)** FBXL5, IREB2, and MFRN2 protein levels in MLE-12 cells was detected using western blotting (*n* = 3). **(F)** MLE-12 cell viability was analyzed using CCK8 (*n* = 5). **(G)** MLE-12 cells were transduced with Mito-Tracker (red), and iron was transduced with Mito-Ferro Green (green) and then imaged using confocal microscopy with Airyscan. Representative images are shown (Scale bar = 5 μm), and boxes mark the enlarged images as shown (Scale bar = 1 μm). **(H)** MLE-12 cells were transduced with Mito-Tracker (red), and DNA was transduced with anti-DNA antibodies (green) and then imaged using a confocal microscopy with Airyscan. Representative images are shown (Scale bar = 5 μm), and boxes mark the enlarged images shown (Scale bar = 1 μm). **(I)** ROS levels were analyzed using a ROS kit (Scale bar = 100 μm). **(J)** The Δψm in MLE-12 cells was measured using JC-1 staining (Scale bar = 100 μm). **(K)** MLE-12 cells were transduced with Mito-Tracker (red), and iron was transduced with Mito-Ferro Green (green) and then imaged using confocal microscopy with Airyscan. Representative images are shown (Scale bar = 5 μm), and boxes mark the enlarged images shown (Scale bar = 1 μm). **(L)** MLE-12 cells were transduced with Mito-Tracker (red), and DNA was transduced with anti-DNA antibodies (green) and then imaged using confocal microscopy with Airyscan. Representative images are shown (Scale bar = 5 μm), and boxes mark the enlarged images shown (Scale bar =1 μm). **(M)** ROS levels were analyzed using an ROS kit (Scale bar = 100 μm). **(N)** The Δψm in MLE-12 cells was measured using JC-1 staining (Scale bar = 100 μm). *P < 0.05, ** *P* < 0.01 and *** *P* < 0.001.

We explored the specific mechanisms underlying EP4 receptor activation in regulating mitochondrial iron deposition. After thalidomide reduced ubiquitination in MLE-12 cells, the effects of EP4 activation on reducing IREB2 and MFRN2 protein levels were offset **(Figure 6A-B)**. This reduction in ubiquitination levels also negated the protective effects of EP4 activation against BLM-induced MLE-12 cell viability decreased **(Figure 6C)**. These data suggest that EP4 activation protects against mitochondrial iron deposition-induced MLE-12 cell dysfunction by promoting the ubiquitination of IREB2.

Additionally, after FBXL5 knockdown in MLE-12 cells, the effects of EP4 activation on IREB2 content and MFRN2 protein levels were abolished **(Figure 6D-E)**. Furthermore, the protective effects of EP4 activation against mitochondrial iron deposition and mtDNA damage were reduced following FBXL5 knockdown **(Figure 6G-H)**. Similarly, FBXL5 knockdown negated the effects of EP4 activation on mitochondrial ROS levels and Δψm **(Figure 6I-J)**. FBXL5 knockdown also negated the protective effects of EP4 activation against BLM-induced MLE-12 cell viability decreased **(Figure 6F)**. These results suggest that decreased FBXL5 expression induces mitochondrial iron deposition and MLE-12 cell dysfunction, whereas EP4 activation protects against mitochondrial iron deposition-induced MLE-12 cell dysfunction by increasing FBXL5 levels.

After MFRN2 overexpression in MLE-12 cells, the effects of EP4 activation on mitochondrial iron deposition and mtDNA damage were hindered **(Figure 6K-L)**. Additionally, the effects of EP4 on mitochondrial ROS levels and Δψm were canceled after MFRN2 overexpression **(Figure 6M-N)**. These findings indicated that EP4 activation protects against mitochondrial iron deposition-induced MLE-12 cell dysfunction by decreasing MFRN2 expression.

These data suggest that EP4 receptor activation improves BLM-induced mitochondrial iron deposition by promoting FBXL5 regulation of the IREB2-MFRN2 axis in MLE-12 cells.

### Activation of the EP4 receptor mitigated AECII mitochondrial iron deposition via FBXL5 regulation of the IREB2-MFRN2 axis in BLM-induced pulmonary fibrosis

**Figure 7:**
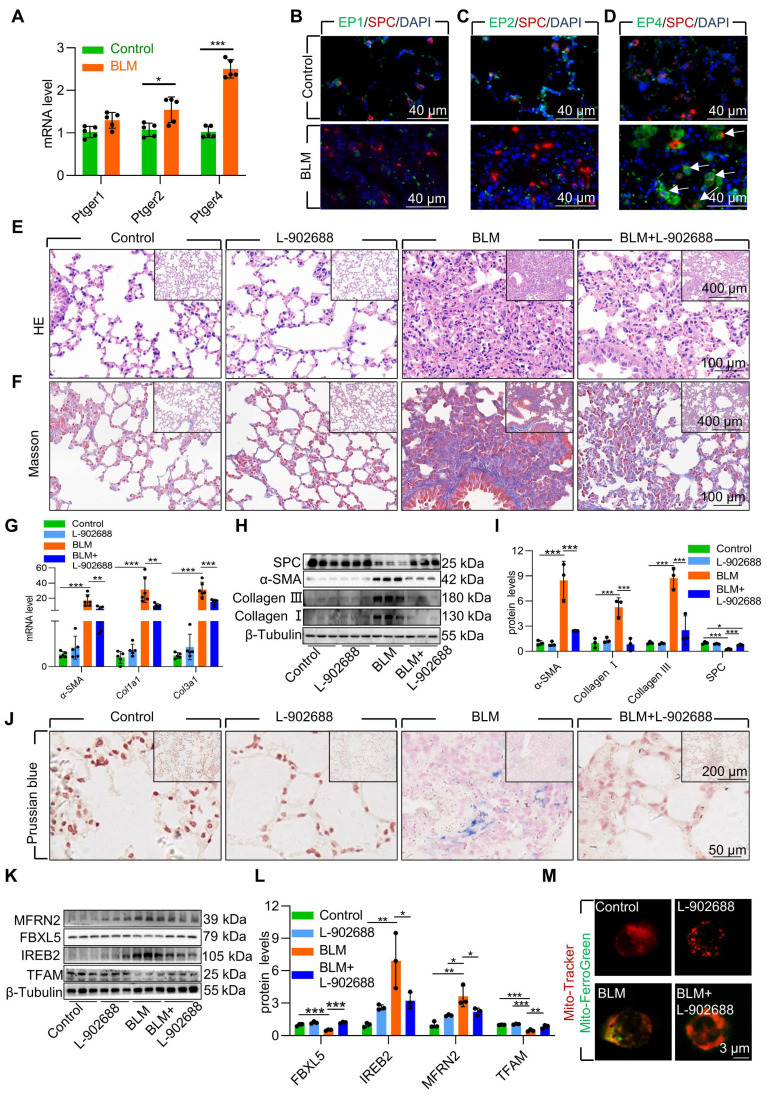
Activation of EP4 receptor mitigated AECII mitochondrial iron deposition via FBXL5 regulation of the IREB2-MFRN2 axis in single-dose BLM-induced pulmonary fibrosis. **(A)** mRNA levels of *Ptger1*, *Ptger2,* and *Ptger4* in the lungs was detected using RT-qPCR (*n* =5). **(B)** EP1 localization in AECII from control and pulmonary fibrosis mice was determined using anti-EP1 antibodies (green) and anti-SPC antibodies (red) (Scale bar = 40 μm). **(C)** EP2 localization in AECII from control and pulmonary fibrosis mice was determined using anti-EP2 antibodies (green) and anti-SPC antibodies (red) (Scale bar = 40 μm). **(D)** EP4 localization in AECII from control and pulmonary fibrosis mice was determined using anti-EP4 antibodies (green) and anti-SPC antibodies (red) (Scale bar = 40 μm). **(E)** Lung histopathological analysis was performed using H&E staining (Scale bar = 100 μm). **(F)** Masson's staining was employed to evaluate collagen disposition (Scale bar = 100 μm). **(G)** mRNA levels of *Acta2*, *Col1a1,* and *Col3a1* in the lungs was detected using RT-qPCR (*n* =5). **(H-I)** α-SMA, Collagen I, Collagen III, and SPC protein levels in the lung was detected using western blotting (*n* = 3). **(J)** Iron deposition in lung tissues was assessed using Prussian blue staining (Scale bar = 50 μm). **(K-L)** MFRN2, FBXL5, IREB2, and TFAM protein levels in the lungs was detected using western blotting (*n* = 3). **(M)** Confocal detection of mitochondrial iron deposition in primary AECII (Scale bar = 3 μm). *P < 0.05, ** *P* < 0.01 and *** *P* < 0.001.

To further validate the role of EP4 receptor activation in the BLM-induced pulmonary fibrosis mouse model, we examined PGE2 receptors in pulmonary fibrosis mouse lung tissues. The results indicated that the EP4 receptor exhibited the highest and most considerable changes in mRNA levels compared with those in mice with pulmonary fibrosis induced by BLM **(Figure 7A)**. Immunofluorescence results also demonstrated elevated protein levels of the EP4 receptor in AECII compared with that in mice with pulmonary fibrosis induced by BLM **(Figure 7B-D)**. These findings suggest that the PGE2-EP4 signaling pathway plays a pivotal role in BLM-induced pulmonary fibrosis in mice.

Subsequently, we observed the impact of the PGE2-EP4 signaling pathway on BLM-induced pulmonary fibrosis in mice by administering L-902688 on day 10 after BLM administration, a key stage in fibrosis progression. Histological examination via HE and Masson's trichrome staining revealed that activation of the PGE2-EP4 signaling pathway ameliorated BLM-induced lung tissue damage and collagen deposition **(Figure 7E-F)**. Moreover, the expression of α-SMA, Collagen I, and Collagen III was substantial reduced in mice exposed to BLM, as confirmed by quantitative real-time polymerase chain reaction (RT-qPCR) and western blotting **(Figure 7G-I)**. Additionally, western blotting revealed that EP4 receptor activation improved the reduced SPC levels induced by BLM, suggesting protection against the loss and damage of AECII in BLM-induced pulmonary fibrosis mice **(Figure 7H-I)**.

To elucidate the mechanism underlying the amelioration of BLM-induced pulmonary fibrosis following exogenous EP4 receptor activation, we examined iron deposition in lung tissues. Prussian blue staining revealed that EP4 receptor activation considerably reduced iron deposition in the lungs of mice with BLM-induced pulmonary fibrosis **(Figure 7J)**. Activation of the EP4 receptor also increased FBXL5 levels, reduced IREB2 content, and decreased MFRN2 levels **(Figure 7K-L)**. Furthermore, EP4 receptor activation increased TFAM levels, protecting mtDNA from damage in mice exposed to BLM **(Figure 7K-L)**. Finally, the effect of EP4 receptor activation on iron deposition in AECII mitochondria was observed in BLM-induced pulmonary fibrosis mice. Primary AECII were extracted using magnetic-activated cell sorting. EP4 receptor activation substantially decreased mitochondrial iron deposition in AECII isolated from the lungs of BLM-induced pulmonary fibrosis mice **(Figure 7M)**.

Overall, exogenous EP4 receptor activation considerably attenuated BLM-induced pulmonary fibrosis by mitigating mitochondrial iron deposition in AECII. Thus, increasing EP4 activity is a potent and effective therapeutic strategy for treating BLM-induced pulmonary fibrosis.

## Discussion

In this study, we observed mitochondrial iron accumulation in AECII in mice with BLM-induced pulmonary fibrosis as well as in BLM-damaged MLE-12 cells. Healthy mitochondria derived from control MLE-12 cells could rescue mitochondrial iron deposition and damage induced by BLM. Furthermore, we found that mitochondrial iron deposition is related to the IREB2-MFRN2 axis, which promotes mitochondrial iron deposition in both MLE-12 cell models and BLM-induced pulmonary fibrosis mouse models. Additionally, FBXL5 was identified as a key player in IREB2 degradation through ubiquitination. This process negatively regulates MRRN2 expression and subsequently reduces mitochondrial iron deposition, providing a new target for pulmonary fibrosis regulation. Moreover, we found that the activation of the EP4 receptor negatively regulates the IREB2-MFRN2 axis by increasing FBXL5 expression, ultimately reducing mitochondrial iron deposition in AECII and potentially offering a treatment strategy for pulmonary fibrosis. Therefore, our findings provide valuable insights into the novel mechanism of AECII damage involved in pulmonary fibrosis.

Iron is an essential element in the body and plays a crucial role in maintaining physiological homeostasis 35. Disruptions in iron metabolism have been linked to the pathogenesis of various diseases 36, 37.

Iron deficiency during early brain development leads to abnormal neural development and cognitive impairment. Conversely, iron accumulation in specific brain regions has been associated with neurodegenerative disorders such as Parkinson's, Alzheimer's, and Huntington's disease 38, 39. In fibrotic diseases, abnormal iron deposition in the hepatocyte-stellate cell axis promotes hepatic fat accumulation and contributes to liver fibrosis 40. In line with these findings, our previous research revealed substantial iron deposition in the lung tissues of both patients and mice models with pulmonary fibrosis, particularly in epithelial cells, and that chelated iron inhibited pulmonary fibrosis, thereby suggesting abnormal iron metabolism plays an important role in the occurrence and development of pulmonary fibrosis 10.

Mitochondria serve as primary cellular hubs for iron utilization. Mitochondrial iron is essential for the biosynthesis of heme and Fe-S clusters, which act as cofactors for enzymes involved in the tricarboxylic acid cycle and respiratory chain complexes, and plays a vital role in many cellular life processes 41. However, compared with traditional iron chelators, mitochondria-specific chelator treatment considerably inhibits the growth and metastasis of human breast cancer cells 18. This suggests that mitochondrial iron metabolism is independent of cytoplasmic iron metabolism, and that maintaining mitochondrial iron metabolism homeostasis is crucial for cellular activities. The study revealed that mitochondrial iron deposition is present in both BLM-induced mouse pulmonary fibrosis AECII and BLM-damaged MLE-12 cells. Hence, there was abnormal mitochondrial iron metabolism in AECII of pulmonary fibrosis.

Limited mitochondrial iron uptake makes it difficult in maintaining normal cellular functions by inhibiting the mitochondrial respiratory chain reaction. However, excessive mitochondrial iron uptake induces mitochondrial dysfunction 42. Mitochondrial dysfunction leads to increased mitochondrial ROS production, which subsequently causes mtDNA damage and releases, and reduced mitochondrial membrane potential, ultimately leading to cellular damage 43-45. Notably, our study demonstrated that healthy mitochondrial transplantation increased TFAM expression, improved mtDNA damage and release, and ultimately rescued damaged mitochondrial dysfunction and cell viability after BLM injury, suggesting that mitochondrial iron deposition-induced mitochondrial dysfunction was involved in AECII damage.

Although there is extensive research on iron metabolism in red blood cells, our understanding of mitochondrial iron metabolism in other cells remains limited 46. Mitochondrial iron levels are complexly regulated by metal chaperones and metal transporters 47, 48. MFRN2 is a major mitochondrial iron porter in nonerythroid cells 49, 50. We observed a considerable upregulation of MFRN2 expression in AECII from fibrotic lung tissue. In addition, MFRN2 overexpression promoted mitochondrial iron deposition and cell damage in MLE-12 cells. Further transplantation of mitochondrial overexpression of MFRN2 into healthy cells resulted in cell damage and loss. Consequently, our findings showed that MFRN2 is a critical player in AECII mitochondrial iron deposition in pulmonary fibrosis.

The iron regulatory proteins (IRP)-iron responsive elements (IRE) complex plays a central role in regulating iron transport-related genes within cells, thereby preventing the degradation of transferrin receptor mRNA by binding to its 3′-UTR and safeguarding it from endonuclease cleavage 30, 51. However, the role of IRP-IRE complexes in regulating mitochondrial iron transporter, especially non-erythrocyte mitochondrial iron transporter MFRN2, remains unknown 52. We identified an interaction between IREB2 and the 3′-UTR of MFRN2. Our results demonstrated a considerable increase in IREB2 content in AECII from pulmonary fibrosis mice and BLM-injured MLE-12 cells. Furthermore, dual luciferase reporter gene experiments confirmed increased interaction between IREB2 and the MFRN2 3′-UTR in BLM-treated MLE-12 cells. This is the first study to highlight the multifaceted role of the IRP-IRE complex, not only in regulating iron transport protein-related genes, but also in modulating the expression of mitochondrial iron transport-related genes and mitochondrial iron levels by regulating MFRN2.

FBXL5 regulates the transcription of iron transport-related proteins by ubiquitinating and degrading IREB2 34. In our study, FBXL5 levels were considerably decreased in AECII from pulmonary fibrosis mice and BLM-injured MLE-12 cells. Additionally, ubiquitination levels of IREB2 were considerably reduced, whereas protein levels of IREB2 were markedly elevated in MLE-12 cells injured by BLM. Furthermore, FBXL5 knockdown substantially increased IREB2 protein levels, upregulated MFRN2 expression, promoted mitochondrial iron deposition, mitochondrial dysfunction, and induced the injury and loss of MLE-12 cells. These findings strongly indicate that FBXL5 is a key target for mitigating mitochondrial iron deposition via the regulation of the IREB2-MFRN2 axis. Therefore, further studies targeting FBXL5 regulation to control AECII mitochondrial iron deposition during lung fibrosis are required.

Although the cyclooxygenase 2/PGE2 signaling pathway is recognized as a classic anti-inflammatory pathway for inhibiting early inflammation and attenuating the progression of pulmonary fibrosis, its action in mid-to-late-stage fibrosis remains uncertain 53. Given that pulmonary fibrosis is often diagnosed at later stages, effective treatment strategies are urgently needed 54. The highest expression of the EP4 receptor was observed in BLM-induced pulmonary fibrosis lung tissues in our study. To assess the impact of changes in EP4 receptor expression on pulmonary fibrosis progression, we specifically activated the PGE2-EP4 signaling pathway 10 days after BLM administration during the fibrosis development period. Our data demonstrated that sustained activation of the EP4 receptor in MLE-12 cells resulted in the upregulated expression of FBXL5, which in turn reduced IREB2-MFRN2 signaling, effectively ameliorating mitochondrial iron deposition, mtDNA damage, mitochondrial dysfunction, and cellular injury induced by BLM. Furthermore, our study showed that EP4 receptor activation considerably increased FBXL5 levels and effectively blocked the IREB2-MFRN2 axis, thereby reducing AECII mitochondrial iron deposition and attenuating fibrosis severity in BLM-induced pulmonary fibrosis mice. Therefore, investigating the specific mechanisms and regulatory strategies of abnormal mitochondrial iron metabolism will contribute to our understanding of the mechanisms underlying pulmonary fibrosis pathogenesis and provide new avenues for therapeutic intervention. Further research is warranted to explore the clinical translation of these findings and to assess the efficacy of targeting mitochondrial iron deposition in patients with pulmonary fibrosis.

This study had some limitations. First, we demonstrated mitochondrial iron deposition in AECII, and confirmed that MFRN2 was involved in BLM-induced pulmonary fibrosis in mice by promoting mitochondrial iron deposition in AECII. However, we did not validate this observation in clinical patients with pulmonary fibrosis. Second, we only demonstrated that MFRN2 is a key factor in the regulation of mitochondrial iron deposition in AECII *in vitro*. However, we did not bidirectionally verify the role of MFRN2 in regulating AECII mitochondrial iron deposition in pulmonary fibrosis of mice with AECII that specifically overexpress or knockdown MFRN2. Finally, efficient and sustained mitochondrial transfer by pioglitazone-Fe-human placental-derived mesenchymal stem cells not only restarted adenosine triphosphate synthesis and reduced cellular oxidative phosphorylation but also re-activated the inhibited mitophagy in injured lung epithelial cells 55, 56. However, in our study, we did not further explore the specific mechanism by which healthy mitochondrial transplantation rescued BLM-damaged MLE-12 cells.

## Materials and Methods

### Animals

Male C57BL/6 mice (eight to ten weeks old) were procured from Hunan SJA Laboratory Animal Co., Ltd. and housed in the animal facility of Central South University. The mice were maintained in a sterile room under 12 h light/dark cycles and provided ad libitum access to food and water. All experiments involving mice were conducted in accordance with the National Institutes of Health Guide for the Care and Use of Laboratory Animals. All animal experiments were approved by the Animal Care and Use Committee of Central South University (Changsha, China, Grant numbers: 2018sydw080).

### Animal models and treatment

Single-dose BLM-induced classical pulmonary fibrosis model: The mice were intraperitoneally injected with pentobarbital (80 mg/kg) prior to surgery. Pulmonary fibrosis was induced in mice by an intratracheal injection of 1.5 mg/kg BLM (Nippon Kayaku, Japan), as in a previous study 57. After 10 d of BLM administration, daily intraperitoneal injection of EP4 receptor activator L-902688 (0.25 µg/kg/d; Cat#C5320; APEXBIO; USA) or saline was used to assess fibrosis progression. On day 21 after BLM administration, the mice were euthanized, and lung tissues were collected and stored at -80 °C for subsequent fibrosis analysis.

Repetitive-dose BLM-induced mouse model that closely resembled the clinical features of pulmonary fibrosis: The mice were anesthetized with ether prior to surgery. After anesthesia, the mice received an intratracheal injection 20 μL of 1 mg/kg/w BLM or normal saline (control) once a week for six weeks 58. On day 42 after BLM administration, the mice were euthanized and lung tissues were collected and stored at -80 °C until analysis of the pending fibrosis plateau.

### Histopathological evaluation

Paraffin embedding: All mice lung tissues were immersed in 4% paraformaldehyde for 12 h at 25 ℃. Subsequently, the tissues were dehydrated through an ethanol gradient (80%, 90%, and 100%) to remove water and embedded in paraffin wax. Sections of 3-μm thickness were cut for analysis.

Hematoxylin-eosin (HE) staining: Dewaxed and rehydrated paraffin sections were subjected to staining for routine pathological examination to assess lung tissue damage.

Masson staining: Dewaxed and rehydrated paraffin sections were subjected to staining with Regaud's hematoxylin dyeing solution to visualize collagen deposition.

### Cell culture

Primary AECII were extracted from CD45^-^/CD326^+^ mice whole lung cells by magnetic bead screening according to the literature 59. The AECII were grown in confocal dishes at a concentration of 1×10^4^ cells/mL and cultured in AECII complete culture medium (Cat# CM-M003; Pricella, China) at 37 °C in 5% CO_2_ for 1.5 d. The mice type II alveolar epithelial cell line MLE-12 was purchased from the Cell Bank of the Chinese Academy of Sciences (Shanghai, China). MLE-12 cells were maintained in Dulbecco's Modified Eagle's Medium (DMEM)/Nutrition Mix F12 (Cat# PM150312; Pricella Life Science & Technology Co., Ltd, China) supplemented with 2% fetal bovine serum (Cat# 164210-50; Pricella Life Science & Technology Co., Ltd, China) and penicillin (100 U/mL)/streptomycin (0.01 mg/mL) at 37 °C in 5% CO_2_. FBXL5 siRNA was loaded into MLE-12 cells by Lip-3000 (Cat#L3000075; Thermo Fisher Scientific; USA) for 48 h to construct FBXL5 knockdown cells. MFRN2 overexpression cells were constructed by loading SLC25A28 overexpression plasmid into MLE-12 cells by NEOFECT™ DNA transfection reagent (Cat# TF201201; NEOFECT; China) for 48 h.

### Mitochondria extraction

Cellular mitochondria were isolated using a Mitochondrial Isolation Kit for Mammalian Cells (cat # 89874; Thermo Fisher Scientific). The cells were initially digested into individual cell suspensions using trypsin and subsequently disrupted by grinding with a glass rod. The mitochondria were then purified from the cytoplasm by gradient centrifugation according to the manufacturer's instructions.

### Mitochondria transplantation

Mitochondrial donor cells were transfected with a pDsRed2-Mito vector (Cos9XTM, China) to specifically label the mitochondria. The cells were broken down by trypsin digestion and cellular mitochondria were extracted using a Mitochondrial Isolation Kit for Mammalian Cells (Cat# 89874; Thermo, USA). The extracted mitochondria were co-cultured with recipient cells at concentrations of 2.5, 5, 10, and 20 μg/mL of protein for 48 h 60. The positive transplantation rate was assessed by using laser confocal microscopy to determine the optimal mitochondrial concentration and transplantation time.

### Cell viability assay

MLE-12 cells were cultured in DMEM/F12 medium supplemented with 2% fetal bovine serum and seeded in 96-well plates at a density of 2000 cells per well. After treatment, Cell Counting Kit-8 solution (CCK-8, Cat# GK10001; GLPBIO, USA) was added to each well according to the manufacturer's instructions. The cells were then incubated at 37 °C for 1-4 h and the cell viability was detected by enzyme labeling at 450 nm light wave.

### Quantitative real-time polymerase chain reaction (RT-qPCR)

Total RNA from MLE-12 cells and lung tissues was isolated using *RNAex Pro* reagent (Cat# AG21101; ACCURATE BIOTECHNOLOGY (HUNAN) CO., LTD., China) and reversed transcribed using a 5× TransScript® Uni All-in-One SuperMix for qPCR (Cat# RR047A; Taraka, Japan) according to the manufacturers' instructions. RT-qPCR was performed using PerfectStart® Green qPCR SuperMix (Transgene, China) and detected with a Bio-Rad RT-qPCR system (CFX96 Touch™, Bio-Rad, USA) 61. Gene expression was quantified using the 2^^(-ΔΔCt)^ method, as outlined in the previous study. The primers used in this study were synthesized by Sangon Biotech (Shanghai, China). The primer sequences are listed in Table 1.

### Western blotting

Proteins from lung tissue or MLE-12 cells were homogenized and lysed in RIPA buffer (Cat# R0010; Solarbio, Beijing, China) supplemented with a Protease Inhibitor Cocktail (Cat# K1019; APEXBIO, USA) 62. The samples were reduced using β-mercaptoethanol and denatured at 90 °C for 5 min. Total protein concentration was determined using the BCA Protein Assay Kit (Cat# P0012S; Beyotime, China). Protein samples were separated on 12% or 10% sodium dodecyl sulfate-polyacrylamide gels and transferred onto 0.22 μm or 0.45 μm polyvinylidene fluoride membranes (Millipore, Bedford, MA). The membranes were blocked with 5% skimmed milk powder and washed with tris buffered saline with tween 20 (TBST) detergent at 4 °C. The procedure was performed with primary antibodies: Collagen I (Cat# 14695-1-AP; Proteintech, China; 1:2000), Collagen III (Cat# 22734-1-AP; Proteintech, China; 1:2000), α-SMA (Cat# 19245; Cell Signaling Technology, USA; 1:2000), FN (Cat# 15613-1-AP; Proteintech, China; 1:2000), β-Tubulin (Cat# 10094-1-AP; Proteintech, China; 1:10000), TFAM (Cat# 22586-1-AP; Proteintech, China; 1:2000), MFRN2 (Cat# orb457153; Biorbyt; United Kingdom; 1:2000), FBXL5 (Cat# A5602; ABclonal; USA; 1:100), IREB2 (Cat# 23829-1-AP; Proteintech, China; 1:2000), Ubiquitin (Cat# 10201-2-AP; Proteintech, China; 1:2000). The membranes were then probed with horseradish peroxidase-linked anti-immunoglobulin (Ig)G secondary antibody (1:5000; Proteintech) for 1 h at 25 ℃. After three washes with TBST, the immunoreactive bands were visualized using an enhanced chemiluminescence reagent (Millipore) and autoradiography. Images were captured using ChemiDoc XRS (Bio-Rad) and quantified using Image Lab 3.0 analysis software (Bio-Rad).

### Co-IP

All samples used for Co-IP were treated with Cell Lysis Buffer for western blotting and IP (Cat# P0013; Beyotime; China) supplemented with a protease inhibitor (Cat# K1019; APEXBIO, USA). Proteins containing IREB2 were precipitated using Protein A/G (Cat# L-1004; Biolinkedin, China) and the ubiquitin levels were detected by western blotting.

### Tissue, cytoplasmic, and mitochondrial iron deposition assays

Prussian blue staining: Paraffin-embedded lung tissue sections (3 μm) were dewaxed and rehydrated. Prussian blue staining was performed following the instructions provided with the Prussian blue staining kit (Cat# G1426; Solarbio, China). Iron deposition in the lung tissue was determined by the formation of a stable blue compound.

Cytoplasmic and mitochondrial iron deposition assays: The cells were first digested with trypsin. Mitochondria and cytoplasm were separated using the mitochondrial extraction kit described above. The iron content was determined by combining iron with Ferene S to form a colored compound. The absorbance at 593 nm was measured using a spectrophotometer, and the iron content was calculated using a standard curve from the iron content assay kit (Cat# ab83366; Abcam; England).

Mitochondrial iron deposition assays: MLE-12 cells were seeded in confocal dishes. Cells were labeled with Mito-Tracker Red (Cat# C1035; Beyotime, Chian). After drug treatment, the iron in mitochondria was labeled in green using Miito-FerroGreen (Cat# M489; DOJINDO; Japan) according to the manufacturer's instructions. Iron deposition in mitochondria was detected using confocal microscopy (ZEISS LSM 900 with Airyscan 2; Carl Zeiss; Germany).

### Mitochondrial functional impairment assay

Extraction of mtDNA: Tissue and cell samples were extracted for total DNA using the DNA extraction kit (Cat# OSR-M401; Tiangen; China). The mtDNA-specific fragments were amplified using ordinary PCR with a concentration of 1000 ng/μL, as determined by concentration measurement. The mtDNA damage was analyzed using agarose gel electrophoresis. The mtDNA primers used in this experiment were synthesized by Sangon Biotech. The primer sequences are shown in Table 1.

MLE-12 cells were seeded in confocal dishes. Cells were labeled with Mito-Tracker Red (Cat# C1035; Beyotime, China). After drug treatment, the DNA in mitochondria was labeled in green using an anti-DNA antibody (Cat# AC-30-10; Progen; Germany) for one night at 4 °C, followed by goat anti-mouse IgM (H+L) FITC antibodies (Cat# E-AB-1067; Elabscience Biotechnology Co., Ltd.; China) for 1 h at 25 ℃. Finally, coverslips were mounted with fluorescent mounting media. The mtDNA release was detected using confocal microscopy (ZEISS LSM 900 with Airyscan 2; Carl Zeiss).

Measurement of Δψm: The Δψmin of MLE-12 cells was detected using the Δψm assay kit with JC-1 (Cat# M8650; Solarbio, China), following the manufacturer's instructions. The cells were incubated with JC-1 working solution for 20 min at 37 °C in the dark. They were then washed twice with JC-1 buffer solution before immunofluorescence analysis. The mitochondrial function was reflected by the ratio of monomer to polymer.

ROS: MLE-12 cells were grown in 48-well plates and treated with the designated drugs. Mitochondria were stained using the MitoSOX™ Red mitochondrial superoxide indicator (Cat# M36008; Invitrogen; USA) following the manufacturer's instructions. The ROS in the mitochondria was measured using fluorescence microscopye.

OCR: MLE-12 cells were grown in 96-well plates and treated with the designated drugs. Extracellular OCR was performed following the instructions provided with the Extracellular OCR Plate Assay Kit (Cat# E297; Dojindo, Japan) 63. The phosphorescence intensity was measured using a multifunctional enzyme marking instrument.

### Immunofluorescence

Paraffin sections were dewaxed and rehydrated. For detection, a tyramine signal amplification-based multiplexed immunofluorescence assay was performed using the triple standard multiplex immunofluorescence Kit (Cat# AFIHC025; AiFang Biological, China). The following protocol was followed:

The cells were fixed in 4% paraformaldehyde for 0.5 h. Then permeabilized with a solution containing 0.2 µL Triton, 0.25 g bovine serum albumin (BSA), and 5 mL phosphate belanced solution (PBS) for 1 h at 25 ℃. The following primary antibodies were used: anti-SFPTC (Cat# PA5-71680; Thermo; USA; 1:50), anti-FBXL5 (Cat# DF14329; Affinity; China; 1:100), anti-IREB2 (Cat# R1706-12; HUABIO, China; 1:100), anti-MFRN2 (Cat# orb457153; Biorbyt; UK; 1:100), anti-DNA (Cat# 690014S; PROGEN; Germany; 1:200), and anti-TFAM (Cat# 22586-1-AP; Proteintech; China; 1:200), anti-EP1 (Cat# A2913; Abclonal; China; 1:200), anti-EP2 (Cat# A9053; Abclonal; China; 1:200) and anti-EP4 (Cat# 24895-1-AP; Proteintech; China; 1:200) were used. Fluorescent secondary antibody for 1 h at 25 ℃ and 4',6-diamidino-2-phenylindole (DAPI) for 15 min at 25 ℃ before measured using fluorescence microscope.

### Statistical analysis

All experiments were independently repeated thrice. Statistical analysis was performed using SPSS 20.0 or GraphPad Prism (GraphPad Software). The following tests were used for comparisons between groups: analysis of variance (ANOVA) with two-tailed unpaired Student's t-test and two-way ANOVA followed by Bonferroni correction for multiple comparison testing. Non-parametric statistical analysis was used for data that were not normally distributed. *P* < 0.05 was considered statistically significant.

## Supplementary Material

Supplementary figures.

## Figures and Tables

**Table 1:** Sequences of specific primers used in this study.

Gene	Forward primer sequence	Reverse primer sequence
*Acta2*	CCAACTGGGACGACATGGAA	TCTGTCAGCAGTGTCGGATG
*Col1a1*	GGGTGATCCCCCTTGAGTAT	GATGCCTCCATTGTGGAAGT
*Col3a1*	GTCTGGTGGCTTTTCACCCT	AGTTCGGGGTGGCAGAATTT
*Cox III -Cytb*	GGAACAACCCTAGTCGAATGAATTTG	GTGGGACTTCTAGAGGGTTAAGTGG
*Cytb-Nd1*	GGCCGCGATAATAAATGGTAAGATG	AACTGATAAAAGGATAATAGCTATGGTTACTTCAT
*FBXL5*	TCTGACCAAGAGACTGGACGA	TCAGCATGAGGACCGTTAATGT
*MFRN2*	CCACTGTCACCACGCACAT	CTTGACGACTTCCGCTGGAT
*Mouse 18S*	AAACGGCTACCACATCCAAG	CCTCCAATGGATCCTCGTTA
*PTGER1*	GTTTTGCCCACTCAAGGCTC	ATATCAGTGGCCAAGAGGGC
*PTGER2*	CACCTTCGCCATATGCTCCTT	TTTCCTAAGCTCCAGCTTCCAG
*PTGER4*	TAGCCTCTCTGGCTTTCCAA	TACCTCCAACCTCAGCCATC
*TFAM*	TCGCATCCCCTCGTCTATCA	CCACAGGGCTGCAATTTTCC

**Table 2:** Target sequences of siRNA.

Name	Sequences (5′ to 3′)
FBXL5 siRNA	CCACTGAACTTGATACTGA
