# Supplementary material for: Abnormal mitochondrial iron metabolism damages alveolar type II epithelial cells involved in bleomycin-induced pulmonary fibrosis
Source: Theranostics. 2024 Apr 15;14(7):2687–705. doi: 10.7150/thno.94072 (PMC11103499; doi:10.7150/thno.94072)
Supplement: Supplementary file 1 — Supplementary figures. [file thnov14p2687s1.pdf]

Supplemental Information

for

Abnormal mitochondrial iron metabolism damages alveolar type II epithelial cells involved in bleomycin-induced pulmonary fibrosis

Min Shao[a], Haipeng Cheng[b], Xiaohong Li[b], Yujia Qiu[a], Yunna Zhang[a], Yanfen Chang[a], Jiafeng Fu[a], Mengxia Shen[a], Xinxin Xu[a], Dandan Feng[a], Yang Han[a], ShaoJie Yue[c], Yan Zhou[a]*, Ziqiang Luo[a,d]*

[a]Department of Physiology, Xiangya School of Medicine, Central South University, Changsha, Hunan 410078, China

[b]Department of Pathology, The Second Xiangya Hospital, Central South University, Changsha, Hunan, 410000, China

[c]Department of Pediatrics, Xiangya Hospital, Central South University, Changsha, Hunan, 410013, China

[d]Hunan Key Laboratory of Organ Fibrosis, Changsha, Hunan, 410013, China

***Corresponding author**

Dr. Yan Zhou or Prof. Ziqiang Luo

Department of Physiology

Xiangya Medical School, Central South University

Changsha, Hunan 410078, China

Tel.: +86-731-82355051

E-mail: zhouyanxy@csu.edu.cn or luoziqiang@csu.edu.cn

Figure S1.

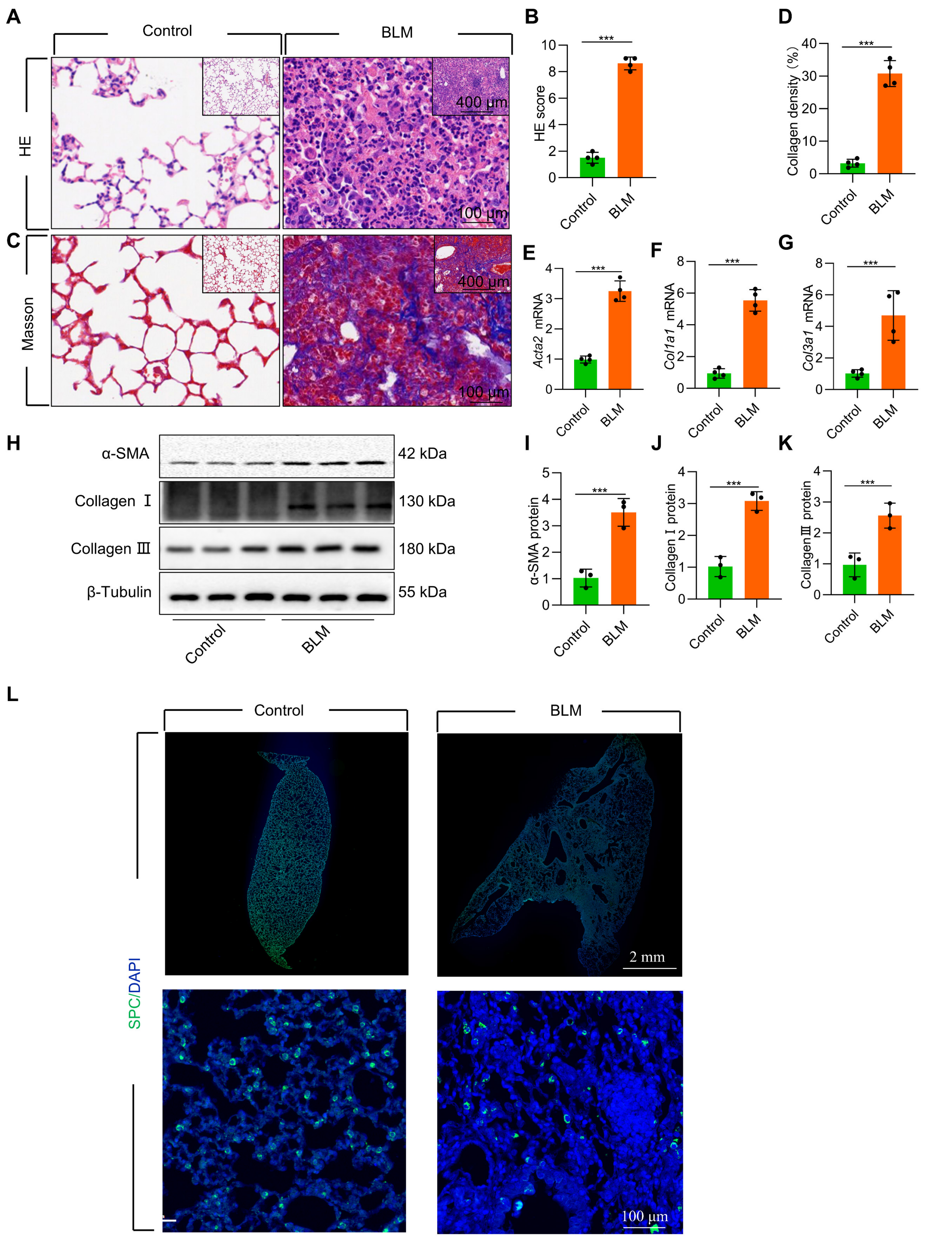

Figure S1. Mitochondrial iron deposition in AECII during repetitive-dose BLM-induced pulmonary fibrosis. **(A)** Lung histopathology with HE staining was performed. **(B)** The HE

score was evaluated by three blinded pathologists (n = 4 per group). **(C)** Masson's trichrome staining was employed to evaluate collagen disposition. **(D)** Quantification of the area occupied by fibrotic stroma, determined by Masson's trichrome staining (n = 4 per group). **(E–G)** mRNA levels of *Acta2*, *Col1a1*, and *Col3a1* in the lungs were detected using RT-qPCR (n = 4). **(H–K)** The protein levels of α-SMA, Collagen I, and Collagen III proteins in the lungs was detected using western blotting (n = 3). **(L)** SPC^+ cells in healthy mice and pulmonary fibrosis mice were detected using an anti-SPC antibody (green) (Scale bar = 100 μm). *** $P < 0.001$.

Figure S2.

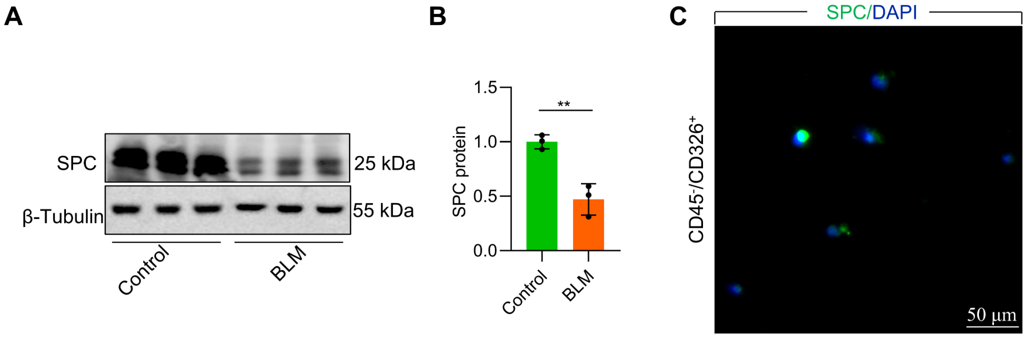

Figure S2. Mitochondrial iron deposition in AECII during single-dose BLM-induced pulmonary fibrosis. **(A–B)** SPC protein levels in the lungs was detected using western blotting (A, B, n = 3). **(C)** SPC protein levels in AECII was determined using anti-SPC antibodies (green) (Scale bar = 50 μm). ** $P < 0.01$.

Figure S3.

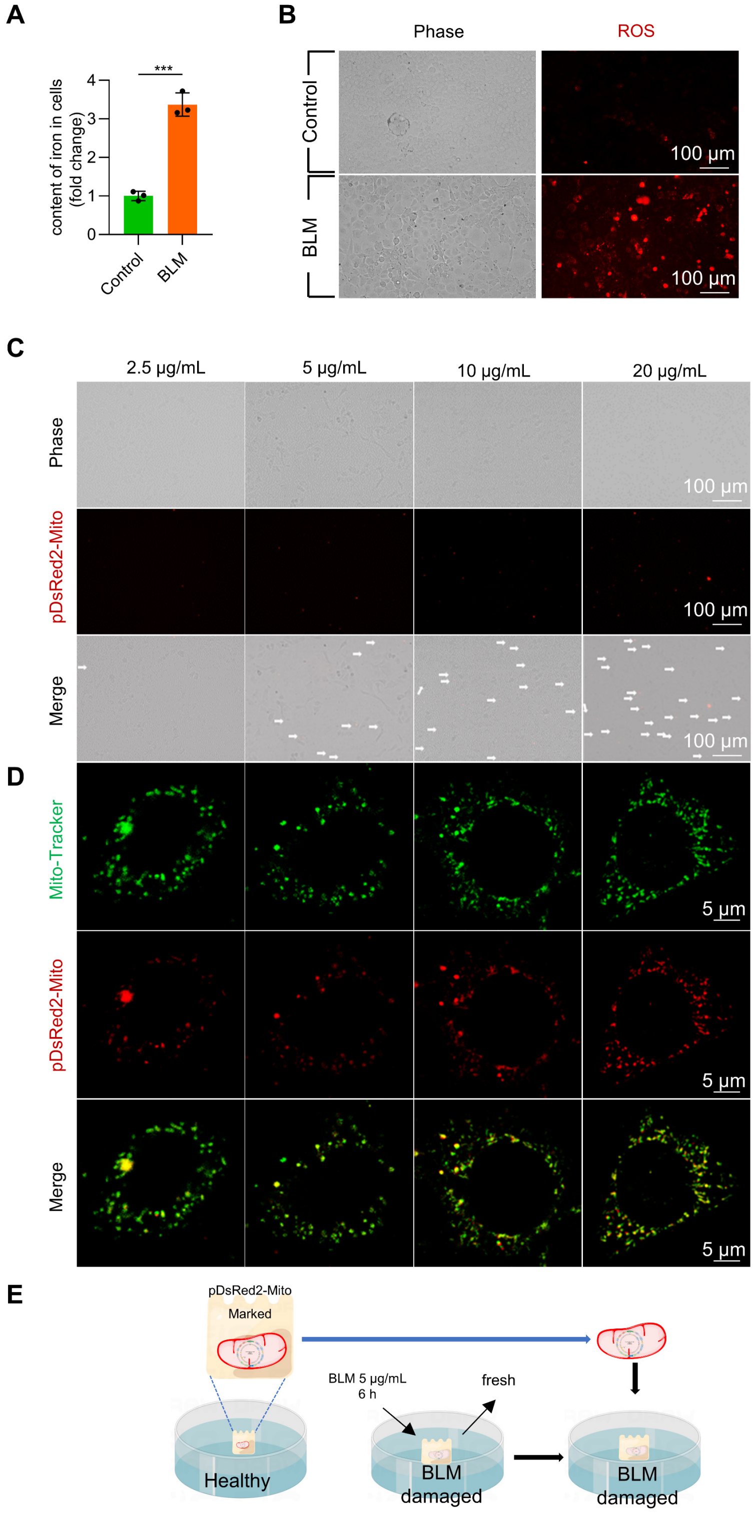

Figure S3. Mitochondrial iron deposition contributed to ME-12 cells injury after BLM-induced damage. **(A)** The cells iron content was analyzed using an iron kit ($n = 3$). **(B)** ROS levels were

analyzed using an ROS kit (Scale bar = 100 μm). **(C)** pDsRed2-Mito-tagged healthy cell mitochondria was cocultured with healthy MLE-12 cells for 48 h. Then, they were detected using immunofluorescence staining (Scale bar = 100 μm). **(D)** pDsRed2-Mito-tagged healthy cell mitochondria was cocultured with healthy MLE-12 cells for 48 h. Then, they were imaged using confocal microscopy with Airyscan. Representative images are shown (Scale bar = 5 μm). **(E)** The MLE-12 cells were pretreated with BLM for 6 hours, after which the BLM was removed and the cells were co-cultured with mitochondria extracted from healthy cells for 48 hours. MLE-12 cells were transduced with Mito-Tracker (red) before being co-cultured. *** $P < 0.001$.

Figure S4.

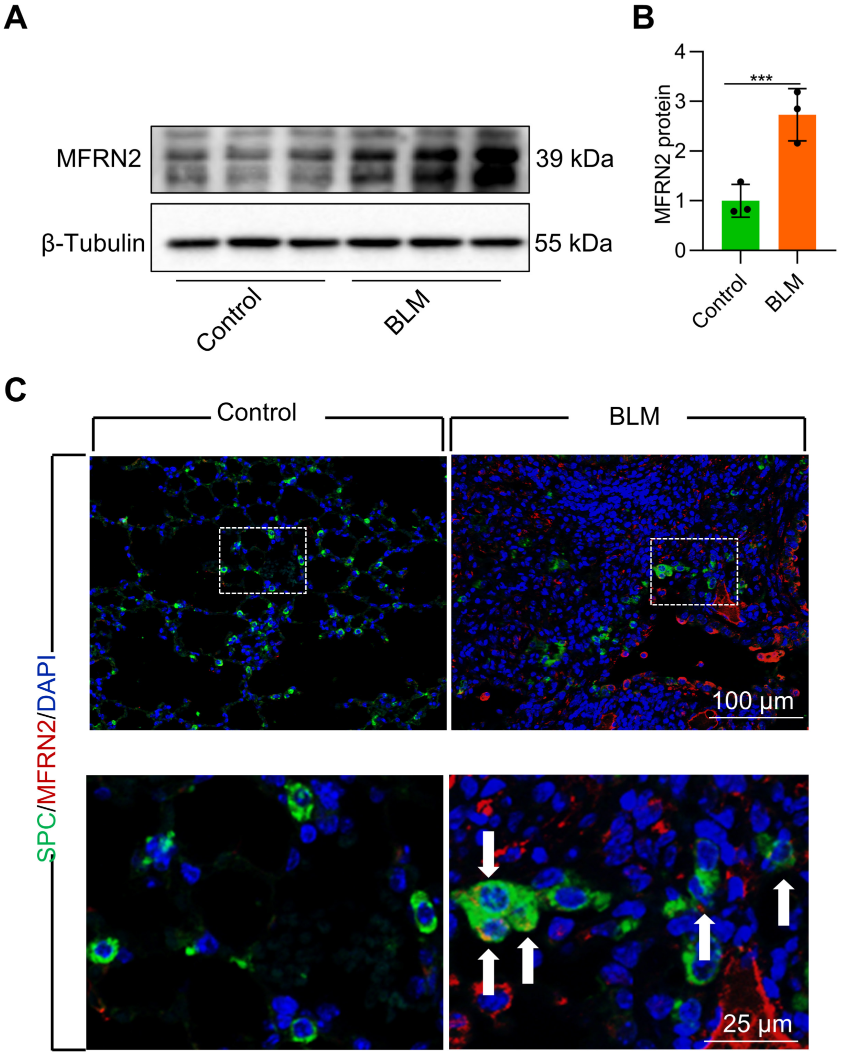

Figure S4. MFRN2 promoted mitochondrial iron deposition in repetitive-dose BLM-induced pulmonary fibrosis. **(A–B)** The protein levels of MFRN2 in the lungs was detected using western blotting (n = 3). **(C)** MFRN2 localization in AECII from control and pulmonary fibrosis mice was determined using anti-MFRN2 antibodies (red) and anti-SPC antibodies (green) (Scale bar = 25 μm). *** $P < 0.001$.

Figure S5.

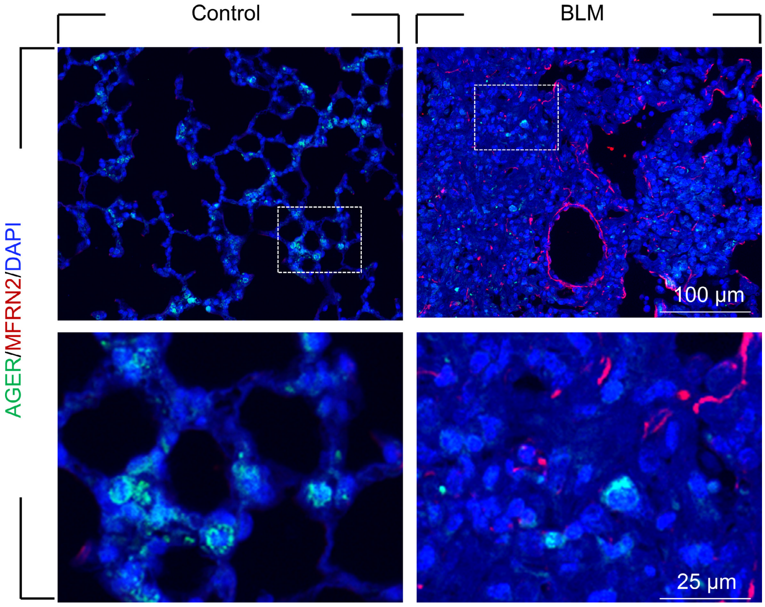

Figure S5. MFRN2 promoted mitochondrial iron deposition in single-dose BLM-induced pulmonary fibrosis. MFRN2 localization in AECI from control and pulmonary fibrosis mice was detected using anti-AGER antibodies (green) and anti-MFRN2 antibodies (red) (Scale bar = 25 μm).

Figure S6.

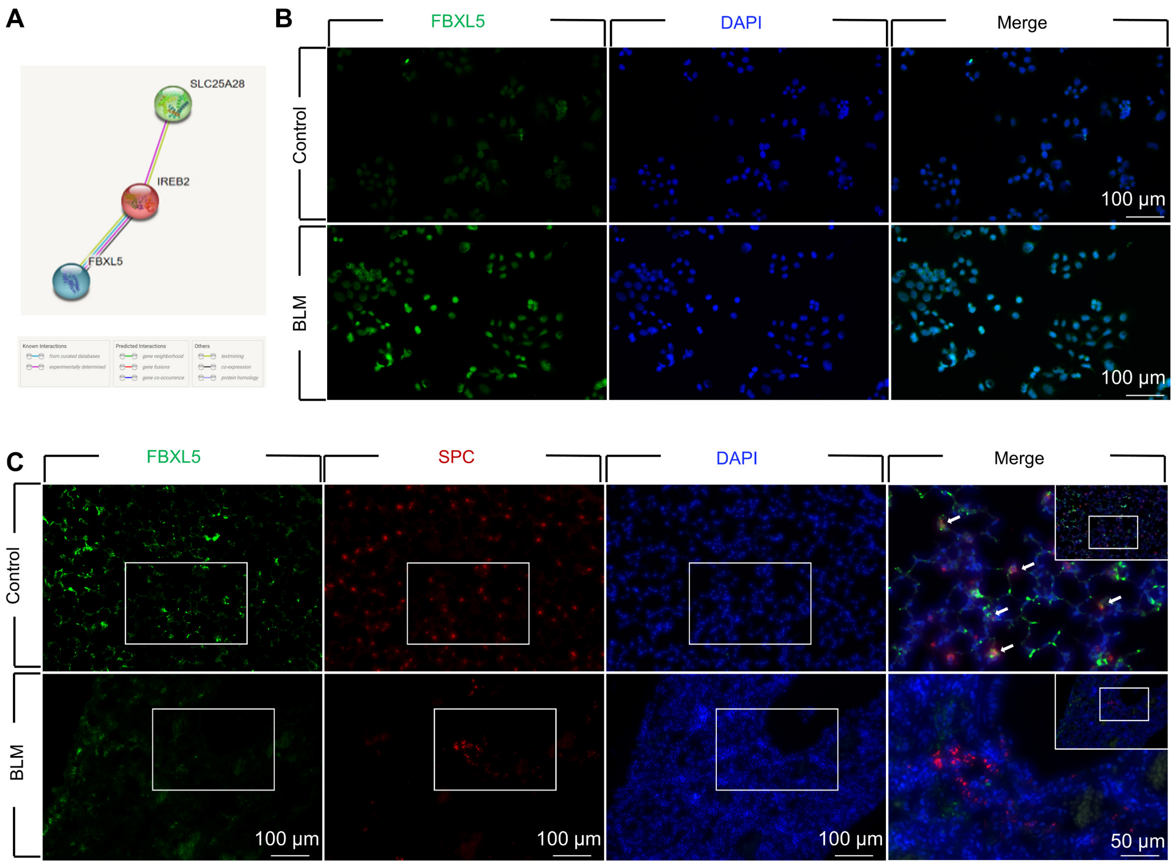

Figure S6. FBXL5 regulated the IREB2-MFRN2 axis, attenuating mitochondrial iron deposition and protecting AECII cells from single-dose BLM-induced pulmonary fibrosis and MLE-12 cells from BLM damage. **(A)** FBXL5 and IREB2-MFRN2 correlation was analyzed by STRING (https://cn.string-db.org/). **(B)** Protein levels of FBXL5 in MLE-12 cells was detected by immunofluorescence staining (Scale bar = 100 μm). **(C)** FBXL5 localization in AECII of the control and pulmonary fibrosis mice using anti-FBXL5 antibodies (green) and anti-SPC antibodies (red) (Scale bar = 50 μm).

Figure S7.

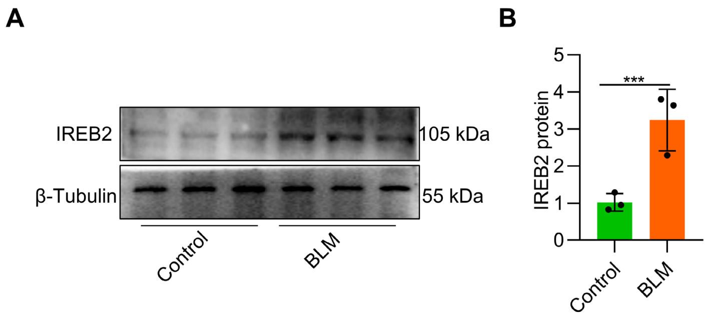

Figure S7. FBXL5 regulated the IREB2-MFRN2 axis, attenuating mitochondrial iron deposition and protecting AECII from repetitive-dose BLM-induced pulmonary fibrosis. **(A–B)** The protein levels of IREB2 in the lungs was detected using western blotting (n = 3). *** P < 0.001.

Figure S8.

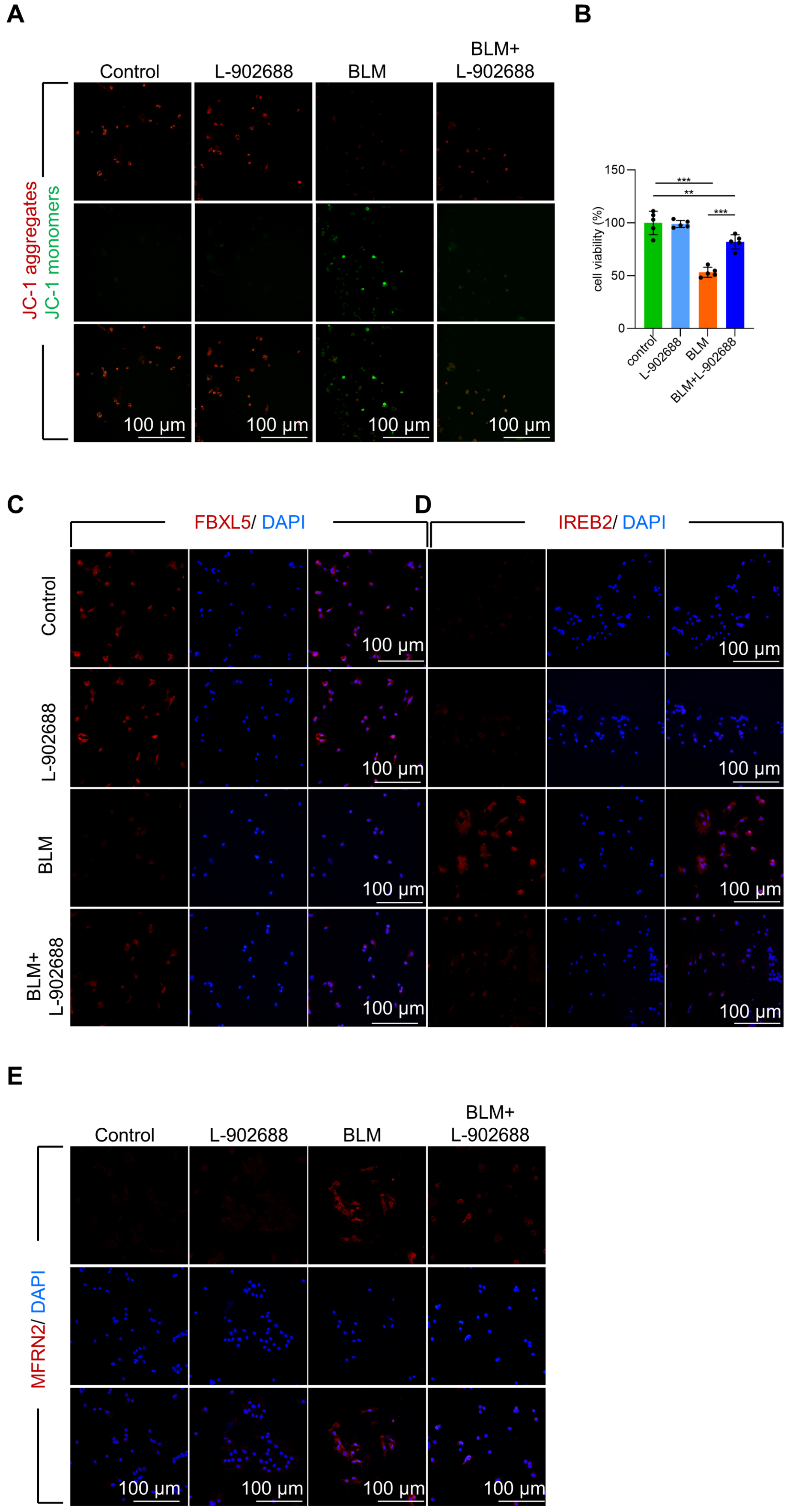
A
B
Control
L-902688
BLM
BLM+
L-902688
JC-1 aggregates
JC-1 monomers
100 μm
cell viability (%)
150
100
50
0
***
**
control
BLM
BLM+L-902688
C
FBXL5/ DAPI
D
IREB2/ DAPI
E
MFRN2/ DAPI

Figure S8. Activation of the EP4 receptor improved BLM-induced mitochondrial iron deposition via FBXL5 regulation of the IREB2-MFRN2 axis in primary AECII. **(A)** The $\Delta\psi m$ in primary AECII was measured using JC-1 staining (Scale bar = 100 μm). **(B)** The primary AECII viability was analyzed using CCK-8 (n = 5). **(C)** FBXL5 protein levels in primary AECII was detected using immunofluorescence staining (Scale bar = 100 μm). **(D)** IREB2 protein levels in primary AECII was detected using immunofluorescence staining (Scale bar = 100 μm). **(E)** MFRN2 protein levels in primary AECII was detected using immunofluorescence staining (Scale bar = 100 μm). ** $P < 0.01$ and *** $P < 0.001$.
